# Contrasting origin of B chromosomes in two cervids (Siberian roe deer and grey brocket deer) unravelled by chromosome-specific DNA sequencing

**DOI:** 10.1186/s12864-016-2933-6

**Published:** 2016-08-11

**Authors:** Alexey I. Makunin, Ilya G. Kichigin, Denis M. Larkin, Patricia C. M. O’Brien, Malcolm A. Ferguson-Smith, Fengtang Yang, Anastasiya A. Proskuryakova, Nadezhda V. Vorobieva, Ekaterina N. Chernyaeva, Stephen J. O’Brien, Alexander S. Graphodatsky, Vladimir A. Trifonov

**Affiliations:** 1 Institute of Molecular and Cell Biology, Novosibirsk, Russia; 2 Theodosius Dobzhansky Center for Genome Bioinformatics, Saint-Petersburg State University, Saint-Petersburg, Russia; 3 Royal Veterinary College, University of London, London, UK; 4 Cambridge Resource Centre for Comparative Genomics, Department of Veterinary Medicine, Cambridge University, Cambridge, UK; 5 Wellcome Trust Sanger Institute, Hinxton, UK; 6 Novosibirsk State University, Novosibirsk, Russia

**Keywords:** B chromosome Evolution, High-throughput Sequencing, DOP-PCR, Siberian Roe Deer, Grey Brocket Deer, Protooncogenes

## Abstract

**Background:**

B chromosomes are dispensable and variable karyotypic elements found in some species of animals, plants and fungi. They often originate from duplications and translocations of host genomic regions or result from hybridization. In most species, little is known about their DNA content. Here we perform high-throughput sequencing and analysis of B chromosomes of roe deer and brocket deer, the only representatives of Cetartiodactyla known to have B chromosomes.

**Results:**

In this study we developed an approach to identify genomic regions present on chromosomes by high-throughput sequencing of DNA generated from flow-sorted chromosomes using degenerate-oligonucleotide-primed PCR. Application of this method on small cattle autosomes revealed a previously described *KIT* gene region translocation associated with colour sidedness. Implementing this approach to B chromosomes from two cervid species, Siberian roe deer (*Capreolus pygargus*) and grey brocket deer (*Mazama gouazoubira*), revealed dramatically different genetic content: roe deer B chromosomes consisted of two duplicated genomic regions (a total of 1.42-1.98 Mbp) involving three genes, while grey brocket deer B chromosomes contained 26 duplicated regions (a total of 8.28-9.31 Mbp) with 34 complete and 21 partial genes, including *KIT* and *RET* protooncogenes, previously found on supernumerary chromosomes in canids. Sequence variation analysis of roe deer B chromosomes revealed a high frequency of mutations and increased heterozygosity due to either amplification within B chromosomes or divergence between different Bs. In contrast, grey brocket deer B chromosomes were found to be more homogeneous and resembled autosomes in patterns of sequence variation. Similar tendencies were observed in repetitive DNA composition.

**Conclusions:**

Our data demonstrate independent origins of B chromosomes in the grey brocket and roe deer. We hypothesize that the B chromosomes of these two cervid species represent different stages of B chromosome sequences evolution: probably nascent and similar to autosomal copies in brocket deer, highly derived in roe deer. Based on the presence of the same orthologous protooncogenes in canids and brocket deer Bs we argue that genomic regions involved in B chromosome formation are not random. In addition, our approach is also applicable to the characterization of other evolutionary and clinical rearrangements.

**Electronic supplementary material:**

The online version of this article (doi:10.1186/s12864-016-2933-6) contains supplementary material, which is available to authorized users.

## Background

B chromosomes are dispensable karyotype elements found in some species of animals, plants and fungi. They seem to originate from duplications and translocations of host genomic regions or result from inter-species hybridization. The number of B chromosomes can vary between individuals and even tissues or cells. They are present in a significant proportion of individuals within populations and are effectively transferred through generations. These features distinguish Bs from other types of supernumerary chromosomes, such as small supernumerary marker [1] or double minute chromosomes [2]. Until recently, little was known about their genetic content, only repetitive elements and high copy number genes such as ribosomal RNA and histone genes were reported [3]. In mammals, duplicated unique protein-coding genes were found on the B chromosomes of canids (red fox and raccoon dog [4–6]) and Siberian roe deer. In the latter case, the copies of genes on B chromosomes were also transcriptionally active [7]. Similar findings were reported in fish, insects and plants (reviewed in [8, 9]).

In Cetartiodactyla, B chromosomes have been described in the Siberian roe deer (*Capreolus pygargus*) [10], grey brocket deer (*Mazama gouazoubira*) [10, 11], red brocket deer (*M. americana*) [10, 11], Brazilian dwarf brocket deer (*M. nana*) [11, 12], small red brocket deer (*M. bororo*) [11, 13], brown brocket deer (*M. nemorivaga*) [14], and Siberian musk deer (*Moschus sibiricus*) [15]. With the exception of an unconfirmed case in the musk deer, these species belong to the subfamily Capreolinae of Cervidae. Our study aims to characterize the B chromosome genetic content in two cervid species: Siberian roe deer and grey brocket deer. In both of these species B chromosomes are among the smallest karyotype elements (only grey brocket deer Y chromosome is tinier). A previous study revealed a 2 Mbp region with three genes (*TNNI3K*, *LRRIQ3* and *FPGT*) on roe deer B chromosomes [7], while B chromosomes of *Mazama* have not yet been studied by molecular methods.

The next generation sequencing (NGS) technologies are actively developing, but their use for the characterization of isolated chromosomes is still quite limited. Whole-genome shotgun sequencing (WGS) with short reads (e.g. Illumina) does not allow the assembly of short DNA fragments into chromosomes directly – this procedure requires long-insert libraries or longer reads (e.g. PacBio) and subsequent anchoring to chromosomes using various genome maps. In B chromosome research, comparative WGS of individuals with and without Bs can be used to identify B-specific blocks of non-repetitive sequence by demonstrating increased read depth in B-carriers [16].

Alternatively, NGS can be also applied to individual chromosomes isolated by flow-sorting or microdissection. These methods usually produce low amounts of DNA (although high-performance sorting is possible [17]) and require subsequent whole-genome amplification (WGA). In our study, we focused on degenerate-oligonucleotide-primed PCR (DOP-PCR) [18] with semi-random primers, a method routinely used for chromosome paint probe construction.

Here, we performed Illumina MiSeq sequencing of flow-sorted B chromosomes of Siberian roe deer [12] and grey brocket deer. Our aim was to analyse the genetic content of mammalian Bs by NGS for the first time and to compare the evolutionary pathways of B chromosomes in two cervid species.

## Results

In this study we analysed B-chromosome-specific DNA derived from flow-sorted chromosomes of Siberian roe deer and grey brocket deer. As controls, we took two samples of chromosome-specific DNA obtained by flow sorting and DOP-PCR amplification from the well-characterized mammalian genomes – dog (*Canis lupus familiaris*) chromosome 12 (CFA12) and a bovine (*Bos taurus*) mixed peak containing chromosomes 23, 26, 28, and 29 (BTA23, BTA26, BTA28, BTA29). Statistics on sequencing and analysis are presented in Tables 1 and 2.

**Table 1:** Statistics of sequencing and mapping of sorted chromosome-specific DNA

Sample	Reference	# reads	Reads, bp	% contam	% target
CFA12	canFam3	746,338	198,383,243	1.3	40.2
BTAMix	bosTau7	708,228	184,977,360	1.1	49.3
CPYB1	bosTau7	1,033,322	304,920,244	1.7	13.8
CPYB2	bosTau7	886,978	268,895,542	1.1	12.7
MGOB	bosTau7	716,158	239,022,974	4.2	31.9

Samples: CFA12 – dog chromosome 12; BTAMix – cattle sorting peak with chromosomes 23, 26, 28, and 29; CPYB1, CPYB2 – Siberian roe deer B chromosomes (technical replicates); MGOB – grey brocket deer B chromosomes. # reads, reads bp – initial number and volume of sequence reads. # contam – percent of reads mapped to human genome (see text for details). # target – percent of reads mapped to target genome with MAPQ > 20 after contamination removal

**Table 2:** Positions occupied by DOP-clones (DOP-positions) and target regions

Sample	Positions (bp) in genome	Target region size, bp	% positions (occupancy) in target regions
CFA12	36,819 (5,285,070)	72,498,081	53.4 (4.4)
BTAMix	50,035 (6,997,158)	202,419,725	82.1 (3.0)
CPYB1	12,158 (1,530,385)	1,979,679	8.3 (14.6)
CPYB2	9,665 (1,182,269)	1,979,679	9.4 (13.2)
MGOB	12,530 (1,934,874)	9,311,710	43.5 (14.3)

Sample names as in previous; Positions (bp) in genome – number of non-repetitive DOP-positions throughout the reference genome and their cumulative size; Target region size, bp – size of regions present on chromosome determined with our method; % positions (occupancy/coverage) in target regions: % positions – percent of DOP-positions in target region relative to the whole genome (column 2), occupancy – percent of target region size (column 3) occupied by DOP-positions

### Properties of chromosome-specific DNA amplified with DOP-PCR

Human DNA is an inevitable source of contamination in high-throughput sequencing experiments, especially in those involving whole-genome amplification from small amounts of starting material, such as microdissected chromosomes [16], ancient DNA [19] or single cells [20]. In our study we adopted an approach used in ancient DNA analyses [19], where reads are aligned to target (dog for CFA12 or cattle for BTAMix, CPYB, and MGOB) and contamination (human) genomes and reads with better alignment to human genome than to target genome are discarded. We used mapping quality as an alignment metric in contrast to initially proposed edit distance, which we found to be prone to alignment length alterations. With this approach we eliminated 1.1–4.3 % of human contamination reads that could affect downstream analysis (Table 1).

In the further search for target regions (i.e. regions corresponding to the sampled chromosomes), overlapping reads were referred as positions corresponding to DOP-PCR amplicons (or DOP-positions). It should be noted that this estimation could be inflated to some extent by non-overlapping reads from ends of longer amplicons. Two peaks of DOP-position sizes were observed for all samples: about 100 bp and 180-200 bp (for example, Additional file 1 Figure S1). Given that we used the Nextera protocol which includes enzymatic DNA fragmentation, it is tempting to attribute the shorter peak to randomly fragmented amplicons and the longer peak to virtually intact ones. We sequenced the DOP-PCR-amplified samples of flow-sorted chromosomes prepared with the TruSeq protocol (based on adapter end-ligation to DNA fragments) and observed only one size peak at about 200 bp (data not included in this study). It is interesting that some amplicon lengths were overrepresented, potentially due to PCR duplicates or DOP-sites located within repeats. Depending on the sample, 9.7 to 50 thousand DOP-positions were recovered throughout the reference genome (Table 2). The fraction of DOP-positions falling in target regions depends on sorting quality, target region size and sequence divergence, e.g. 82 % of positions belong to 202.4 Mbp of target chromosomes in the mixed cattle chromosome sample while only 8–9 % belong to the 2.0 Mbp B chromosome-specific region in the Siberian roe deer (Table 2). DOP-positions cover 3–4 % of target region sequences for autosomes and 13–15 % for B chromosomes (1 position per 3.6–4.9 kbp and per 1.7–2.2 kbp, respectively).

### Automation of target region detection

Isolated chromosome sequencing data are known to be contaminated with whole-genome DNA to some extent [21, 22]. We aimed to develop a method that discriminates target regions corresponding to sampled chromosomes from this noise.

**Fig. 1:**
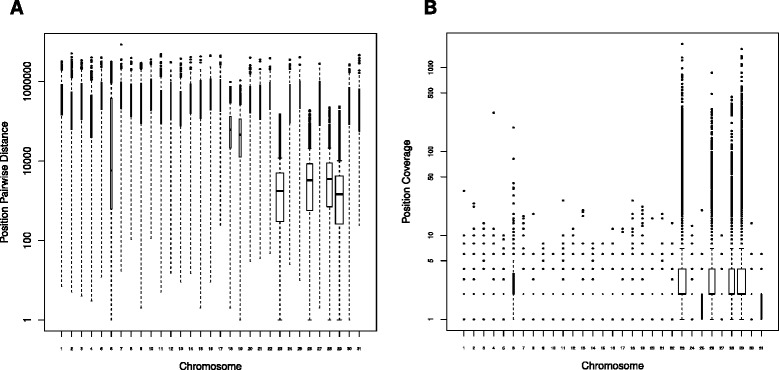
Mean pairwise distances between consecutive DOP-positions (**a**) and position coverage with reads (**b**) in the flow sorting peak containing bovine chromosomes 23, 26, 28, and 29. Note the signal on chromosome 6 corresponding to *KIT* region translocation to BTA29

First, we took samples from species with well-assembled reference genomes – dog chromosome 12 (CFA12) and a mixed peak (BTAMix) containing bovine chromosomes 23, 26, 28, and 29 (BTA23, BTA26, BTA28, and BTA29). Upon visual inspection of read alignments in the UCSC genome browser, we derived two statistics suitable for detection of target chromosomes: pairwise distances between consecutive DOP-positions and read coverage inside DOP-positions. We collected and summarized these statistics for every chromosome in the reference genomes (Fig. 1 for cattle mixed peak). Pairwise distances were significantly lower in the target chromosomes, while read coverage exhibited more outlier high values without significant changes of median values. We also identified additional enrichment peaks corresponding to chromosomes similar in size and CG content (i.e. located closely in the flow karyotype): for CFA12 we observed minor peaks at CFA14 and CFA22, which were expected from sorting [23], for BTAMix – BTA18, BTA19. The cattle mixed peak sample was evaluated with fluorescence *in situ* hybridization (FISH), which yielded signals on the chromosomes identified by sequencing (Additional file 1: Figure S2).

**Fig. 2:**
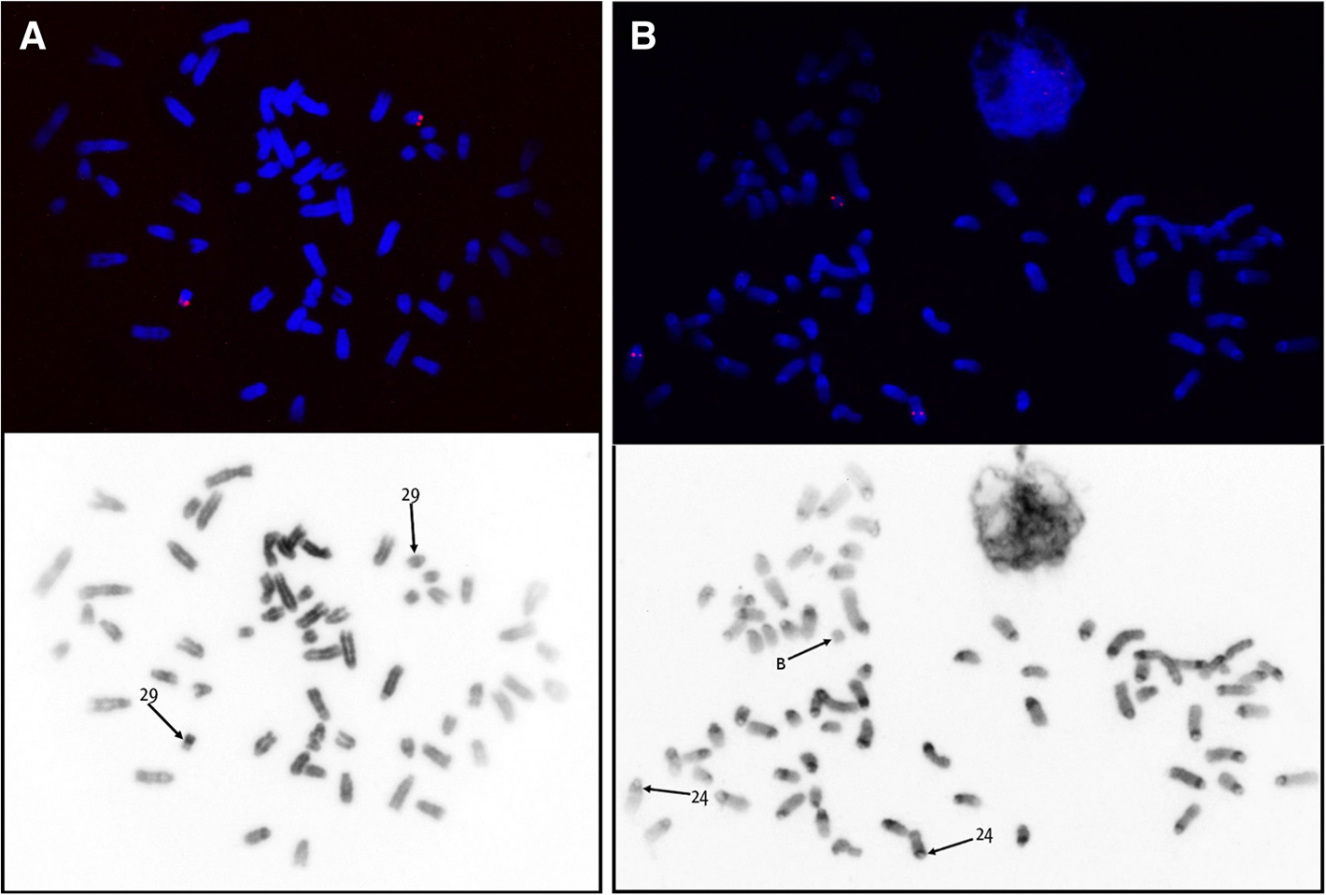
Localization of canine BAC clone with KIT gene on chromosomes of (**a**) cattle and (**b**) grey brocket deer. Note that in brocket deer, autosomal copies of *KIT* are located on MGO24 corresponding to a region of BTA6 [27]. Translocation of a 480-kbp regions from BTA6 to BTA29 is a previously characterized rearrangement associated with colour sidedness in cattle [24]

We then wrote an R script dividing the entire genome into regions with differing mean values of pairwise distances between consecutive DOP-positions (PD) (see Methods for the procedure), where low values of mean PD are characteristic for target regions and higher values – for whole-genome contamination. To test the performance of this method we first analysed control libraries CFA12 and BTAMix (Additional file 2). The script tended to identify multiple regions within target chromosomes, but all of these regions had the lowest mean PD as expected. In the case of the mixed bovine chromosome sample, a relatively small region at BTA6:72,525,912–73,007,603 was detected as target. This region includes the entire *KIT* gene, and this rearrangement was previously described as associated with colour sidedness [24]. Localization of a canine BAC clone with the *KIT* gene on cattle chromosomes confirmed the translocation of this region to one of these small autosomes (Fig. 2a).

### B chromosomes of the Siberian roe deer

Siberian roe deer B chromosomes were readily separated from other chromosomes during sorting due to their relatively small size and high GC content [25]. We sequenced and analysed two samples (CPYB1 and CPYB2) from independent collections in one sorting experiment. Target regions detection in both samples yielded two regions at BTA3:74.55–76.49 Mbp and BTA28:11.36–11.40 Mbp (Table 3, Additional file 2) as well as a small 6–8 kbp artefact region (12 and 16 positions in CPYB1 and CPYB2, respectively) at the end of BTA10 resulting from spurious mapping of telomeric DNA reads. Margins of the larger region on BTA3 were previously estimated with B-specific cDNA sequencing and bovine BAC clone localizations [7]. Our sequencing results provided higher resolution and also indicated that the region had two putative deletions, i.e. regions not covered by reads (74.88–75.30 Mbp and 75.68–75.82 Mbp) (Fig. 2). The first deletion was previously found to be located on the Siberian roe deer Bs by bovine BAC clone mapping, thus it may be a result of DOP-amplification bias or a mapping problem. The second deletion coincides with a cattle genome assembly gap. As previously shown, the BTA3:74.55–76.49 Mbp region includes RefSeq genes *LRRIQ3*, *FPGT* and the 5’ part of *TNNI3K*. The 42-kb region on BTA28 is located between genes *CHRM3* and *ZNF33B*.

**Table 3:** Regions detected on B chromosomes of Siberian roe deer and grey brocket deer with flow-sorted chromosome sequencing

Region	Genes
Siberian roe deer (*Capreolus pygargus*)	
chr3:74,548,027-76,486,586	*LRRIQ3*, *FPGT*, *TNNI3K* (f)
chr28:11,359,721-11,400,841	-
Grey brocket deer (*Mazama gouazoubira*)	
chr1:80,557,678-80,815,945	*LPP* (f)
chr1:132,195,520-132,248,825	*PIK3CB* (f)
chr3:53,939,956-54,035,661	*GFI1*
chr3:97,346,492-97,530,439	*ACOT11* (f), *SSBP3* (f)
chr5:112,038,039-112,092,762	*CCND2*
chr6:67,475,289-69,302,666	*COX7B2*, *GABRA4*, *GABRB1* (f?), *LOC536190* (f?), *ATP10D*, *NFXL1*, *CNGA1*, *NIPAL1*, *TXK*
chr6:72,725,708-72,802,304	*KIT* (f)
chr6:101,762,919-101,983,089	*COQ2*, *HPSE*, *MIR2446*, *MRPS18C*, *FAM175A* (f)
chr6:115,148,215-115,688,073	*MIR2448*, *FBXL5*, *CD38* (f?), *FGFBP1*
chr7:11,112,904-11,187,619	*MAN2B1* (f)
chr7:21,377,048-21,645,864	-
chr8:78,145-782,808	*ANXA10*, *MIR2466*
chr10:28,363,651-29,133,083	*TMCO5B*
chr14:5,823,596-6,377,396	*KHDRBS3* (f)
chr17:46,546,364-46,775,437	*MBD3L1*, *CHFR*, *ZNF268* (f)
chr19:59,129,525-59,366,644	*CDC42EP4*, *FAM104A*, *COG1* (f)
chr22:1,765,826-1,920,639	*EOMES* (f)
chr22:11,626,319-11,984,586	*ACAA1*, *MYD88*, *OXSR1*, *XYLB*, *ACVR2B*, *DLEC1* (f)
chr23:8,994,141-9,084,277	*C23H6orf106* (f), *SNRPC* (f)
chr23:50,678,412-50,701,238	*SERPINB9*
chr25:37,085,021-37,276,232	-
chr25:41,480,442-41,787,784	-
chr28:628,155-915,003	*TRIM67*, *FAM89A*, *ARV1*, *TTC13* (f)
chr28:12,110,619-12,174,951	*RET*
chr29:34,728,621-35,503,130	*OPCML* (f)
chr29:49,338,779-50,252,639	*DHCR7* (f?), *NADSYN1*, *CARS* (f)

Coordinates in cattle genome assembly (bosTau7) are given. Only RefSeq cattle genes are listed, (f) – partial gene fragments located at region margins, (f?) – genes overlapping putative deletions not covered by reads

### B chromosomes of the grey brocket deer

Similarly to the roe deer Bs, the grey brocket Bs were resolved by flow cytometry into a distinct peak [26]. The specificity of the flow-sorted B chromosome DNA was evaluated by FISH (Additional file 1: Figure S3). The sequence analysis of the sample revealed 26 regions of significant homology in the cattle genome, ranging from 23 to 1827 kbp in size (Table 3). Of these regions, three could not be detected by our script due to their small size (23, 55, 90 kbp), but were obvious upon visual inspection of the pairwise distance plot (Additional file 2). This pattern differs dramatically from the one observed in the roe deer, where only two homology regions (neither of which was detected in the grey brocket deer) were discovered. The total size of the regions present on brocket deer Bs was 9.31 Mbp or less due to 1.03 Mbp in 10 putative deletions inferred from long fragments lacking positions covered by reads. According to flow sorting and cytological data, Bs are among the smallest chromosomes in the grey brocket deer karyotype (only Y chromosome is smaller), approximately 10 Mbp in size. Taken together, these findings indicate a lack of extensive amplification of unique genomic regions in grey brocket deer Bs.

To test the target regions observed from sequencing data we localized 9 bovine BAC-clones (Additional file 1: Table S1). For the region BTA10:28.36–29.13 Mbp two BAC clones were hybridized: CH240–472E2 covered the left margin of the region, and showed hybridization signals on B chromosomes (Additional file 1: Figure S4); and CH240–449 J24 (located 50 kbp away from the region detected by sequencing) did not show any FISH signal on B chromosomes. For BTA29:49.34–50.25 Mbp three BACs (CH240–374 L11, CH240–244 F2, and CH240–235P20) located within the region and the BAC CH240–39 K2 overlapping the left margin of the region all showed strong hybridization signals on Bs. For BTA7:21.38–21.65 Mbp two BACs not overlapping with the target region were used and no hybridization signals were observed as expected. The canine BAC clone containing the *KIT* gene gave signals on B chromosomes and on a pair of autosomes (most likely MGO24 according to the roe deer painting results on bovine chromosomes and the presumed conservation of 70-chromosomal karyotype in cervids [27]) (Fig. 2b).

We also performed total cDNA enrichment using the selection of hybrids by affinity capture with sorted chromosome-specific DNA of B chromosomes [7]. Sanger sequencing of 17 clones yielded 5 repetitive sequences, 3 clones originated from BTA29:49.34–50.25 Mbp region, 2 clones – from BTA7:21.38–21.65 Mbp region and BTA10:28.36–29.13 Mbp each, 1 clone from BTA23:8.99–9.08 Mbp. One clone originated from a non-specific region and two clones could not be localized. The remaining single clone was located on the non-chromosomal 65-kbp scaffold Un_JH125058. Upon visual inspection in the genome browser this scaffold showed a position density characteristic of the target region. Orthologous alignment to the human genome (chain/net track from UCSC browser) indicated scaffold homology to Hsa4:46,58–46,66 Mbp. Coordinates of neighbouring sites in the human genome were transferred to the cattle genome by LiftOver which allowed us to attribute the scaffold (reverse complement) to the assembly gap at BTA6:67,529,404–67,612,958. Thus, we were able to include this scaffold in the previously detected BTA6:67.48–69.30 Mbp region on the grey brocket deer Bs.

A total of 34 complete and 21 partial RefSeq genes were found on grey brocket deer B chromosomes (Table 3). Among these, the *KIT* gene was previously found on the B chromosomes of three canid species: red fox and Chinese and Japanese raccoon dogs [4–6]. The *RET* gene was found only in some B chromosomes in the Chinese raccoon dog [6]. The margins of the *KIT* region present on the brocket deer Bs were not identical to those in the cattle chromosome 29 and in the B chromosomes of canids (the latter also included the neighbouring gene *KDR*).

We performed functional enrichment clustering analysis for both complete and complete + partial gene lists. Enrichment signals were not very strong, both gene lists yielded functional clusters associated with ATP-binding/kinase (score 1.33 and 1.30), transit to mitochondria (0.81 and 0.45), cell cycle (0.68 and 0.35), Zn-ion binding/Zn-finger (0.5 and 0.66), and membrane (0.39 and 0.26). Complete + partial genes were also enriched with the cell proliferation/differentiation (1.32) and positive regulation of protein kinase activity (1.00), but such an association disappeared in the list containing only complete genes.

### Sequence variation on B chromosomes

Using sequencing data it became possible to analyse the variants specific to B chromosomes. To get a robust estimate of B chromosome variation patterns we had to ensure that the DOP-PCR protocol does not introduce significant biases into the amplified DNA nucleotide composition. Also, it was necessary to discriminate B-specific variants from sequence divergence between cattle and deer genomes.

The mixed peak of cattle chromosomes 23, 26, 28, and 29 was used to establish variant calling methodology and to ensure that it worked properly. The statistics of the called variants (variant density, heterozygosity, and proportions of different variant types – see Table 4) were similar to the values obtained in SNP discovery by genome resequencing in cattle [28, 29]. We then compared our callset to known SNPs from bovine dbSNP 138 [30]. 12,367 of 19,188 (64.4 %) variants detected from sequencing were novel compared to that dataset. We hypothesized that novel variants with a low read coverage could result from various artefacts, such as sequencing errors, presence of contaminant DNA etc. We applied read depth filters and observed 6,662 novel variants of 10,052 total (66.3 %) at minimum depth 4 and 1,939 of 3,247 (59,7 %) at minimum depth 6. Without significant decrease in number of novel variants (potential errors), we decided to use the entire callsets, but to treat individual variants with caution.

**Table 4:** Sequence variation of DOP-PCR amplified chromosome-specific DNA relative to bosTau7 genome classified relative to bovine RefSeq genes

	BTAMix	CPYB	CPYB-CCA	MGOB
A				
Total	6,068,678	344,775	297,042	1,332,441
Intergenic	4,518,518	262,448	226,421	991,640
Intron	1,501,565	80,987	69,282	327,012
5` UTR	1,337	0	0	27
3` UTR	15,213	0	0	2,780
Coding	32,045	1,340	1,339	10,982
B				
Total	19188 (6374)	15267 (2915)	3086 (1929)	46592 (1735)
Intergenic	14829 (4970)	12187 (2349)	2476 (1530)	35292 (1334)
Intron	4556 (1474)	3134 (558)	605 (392)	10102 (320)
5` UTR	4 (2)	-	-	2 (0)
3` UTR	49 (18)	-	-	101 (8)
Coding syn.	46 (8)	17 (3)	1 (1)	113 (2)
Coding non-syn.	44 (15)	26 (10)	12 (9)	103 (12)
C				
Total	316 (952)	23 (118)	96 (154)	29 (768)
Intergenic	305 (909)	22 (112)	91 (148)	28 (743)
Intron	330 (1,019)	26 (145)	115 (177)	32 (1,022)
5` UTR	334 (669)	-	-	14 (-)
3` UTR	310 (845)	-	-	28 (348)
Coding	356 (1,393)	31 (103)	103 (134)	51 (732)

A. Lengths of cattle genome regions covered by reads. Samples: BTAMix – mixed peak of cattle chromosomes 23, 26, 28 and 29; CPYB – Siberian roe deer B chromosomes (combination of samples CPYB1 and CPYB2); CPYB-CCA – same, but regions not covered by European roe deer contigs are excluded; MGOB – grey brocket deer B chromosomes

B. Number of total and heterozygous (in brackets) variants called and their annotation. Numbers of variants do not add up due to overlapping annotations and excluded NMD_target_transcript annotation. Intergenic variants also include up/downstream variants; coding non-synonymous variants include missense, stop codon gain/loss, frameshift, inframe indels. Sample descriptions – see 3A, except for CPYB-CCA – CPYB variants excluding the variants observed in *Capreolus capreolus* genomic contigs

C. Variant density (bp per 1 variant) calculated as length of sequence covered by reads divided by number of called variants. Numbers are given for all and heterozygous (in brackets) variants. Sample descriptions – see 3B

Sequence variation in interspecies alignment of B chromosomes is hard to interpret, as it can be attributed to the divergence of either orthologous sequences between lineages or paralogous sequence duplicates during B chromosome evolution. In the case of the Siberian roe deer we took advantage of the recently sequenced genome of the closely related European roe deer (*Capreolus capreolus*) [31], which is also known to have incomplete reproductive isolation from the Siberian species [32–34]. The genome was assembled only to contigs with N50 = 10,813 kbp, thus we aligned these contigs to the bovine genome and called all variants in uniquely mapped contigs, obtaining a set of derived positions of European roe deer relative to cattle genome. We subtracted the resulting variants from the variants conventionally called from Siberian roe deer B chromosomal reads (CPYB) unified from two samples CPYB1 and CPYB2. The resulting variants (denoted 'CPYB-CCA' in Table 4) were more B-chromosome-specific, although they still included population-level differences. Several peculiarities of this reduced callset were noted: the variation density remained high (1 variant per 96 bp compared to 1 variant per 316 bp in bovine autosomes), many variants were heterozygous (about 2/3 of heterozygous variants remained in the reduced callset, heterozygous to homozygous variation ratio was equal 1.48 – much higher than 0.50 in bovine autosomes), most of CPYB-CCA variants in protein-coding regions were non-synonymous and heterozygous. The high level of sequence divergence and the extent of protein-disrupting variants were interpreted as indicators of the pseudogenisation process, while the high level of heterozygosity could have resulted either from the previously discovered amplification of protein-coding genes on roe deer B chromosomes or from differences between the 8 Bs present in the karyotype of the cell culture sample [7].

To our knowledge, no brocket genomes have been sequenced yet, thus we could not apply the variant filtration strategy implemented for roe deer. We could only extrapolate some observations from the Siberian roe deer Bs. First, heterozygosity of grey brocket deer B chromosomes was lower than that for the Siberian roe deer Bs: 1 variant per 768 bp compared to 1 per 118 for all roe deer B variants and 1 per 154 for CPYB-CCA variants. In fact, it was closer to the heterozygosity level detected in bovine autosomes (1 variant per 952 bp). This observation together with the larger size of non-repetitive genomic regions compared to roe deer Bs supports the hypothesis for the lack of gene amplification in grey brocket deer Bs, proposed on the basis of their estimated size. Second, no bias towards non-synonymous variants in protein-coding regions was observed, in contrast to roe deer Bs (Table 4B), which we interpret as weak evidence for the lack of pseudogenisation. Similar conclusions were made from the slightly decreased density of derived positions relative to the cattle genome (1 per 29 bp in grey brocket deer Bs versus 1 per 23 bp in Siberian roe deer Bs). Thus, in contrast to the Siberian roe deer, B chromosomes of the grey brocket deer appear to be composed of non-amplified and presumably more conserved duplicated regions.

### Repetitive DNA analysis

We adopted a strategy for repeat characterization by read clustering previously widely used in isolated plant chromosome studies [21]. For the mixed cattle chromosome sample, the content of repetitive DNA identified in read analysis with RepeatExplorer [35, 36] differed significantly from the one obtained from RepeatMasker annotation of cattle chromosomes (Additional file 1: Table S2). RepeatExplorer reported a higher proportion of unannotated repeats and centromeric satellite DNA, which was as expected due to unassembled centromeres in the cattle genome. Other repeat families were mostly under-represented in RepeatExplorer output, sometimes drastically, e.g. 0.08 % versus 2.29 % for LINE L2. A notable exception was a SINE Alu repeat detected only in sorted chromosome samples, evidently resulting from contamination with human DNA. The total percentage of repeats was similar for both methods. These observations imply that the results of RepeatExplorer analysis of DOP-PCR amplified samples sequencing are not directly comparable to the RepeatMasker annotation of the assembled chromosomes.

Thus, we chose to compare RepeatExplorer results between sequences of cattle autosomes and B chromosomes and came to several conclusions (Additional file 1: Table S2 and S3). First, in both roe deer and brocket deer, B chromosomes bear a higher proportion of repeats compared to bovine autosomes. Second, annotation results were reproducible between two independent amplifications of roe deer B chromosomes. Repeat family composition of grey brocket deer B chromosomes was similar to cattle autosomes, while the largest repeat clusters from Siberian roe deer Bs were often unannotated or comprised of low complexity and satellite repeats (Additional file 1: Table S3). In brocket deer Bs, of interest is the cluster 5 that contained virtually intact copies of LTR retrotranspons from the ERVK family, indicating a recent repeat expansion probably due to retroviral infection (Additional file 1: Table S3C).

## Discussion

### NGS of isolated chromosomes

Several aspects are important in the design of the chromosome studies with NGS: DNA source (whole genome or isolated chromosome), isolation method (flow sorting or microdissection), amplification (none or DOP-PCR or MDA). All of these affect the bioinformatic analysis in the ways discussed below.

The supernumerary nature of B chromosomes allows for whole-genome shotgun sequencing (WGS) of individuals with and without B chromosomes, and B-specific blocks can be identified by the increased read depth upon mapping to the reference genome [16]. This method provides high resolution, but sequence variation and repeat composition are hard to interpret because of the main genome background.

The usage of isolated chromosomes for sequencing overcomes most of the WGS problems, but it also has several drawbacks. Two methods for chromosome isolation have been extensively used: flow sorting and microdissection. Flow sorted chromosome-specific DNA proves to be highly efficient for the detection of chromosomal rearrangements by molecular cytogenetics techniques [37, 38] and array-comparative genome hybridization [39, 40]. However, the sorting method has several drawbacks: an inability to separate chromosomes of similar size and GC-content, e.g. human chromosomes 9–12 [41] or cattle chromosomes 23, 26, 28, 29 in our study; contamination with whole-genome and organelle DNA during sample preparation which inevitably involves breaking up millions of cells. Chromosome microdissection in principle produces cleaner results, but most successful experiments yield only several chromosome copies. Our results for flow-sorted chromosomes indicated that while chromosomes within the peak were not readily separated, discrimination from whole-genome background was feasible. Contamination with human DNA is considerable, but can be removed during read mapping, and thus affects mostly repeat analysis – Alu repeats, the major component of the human genome, formed a cluster of up to 2 % of all reads.

Only high-throughput sorting produces amounts of DNA suitable as input for NGS. Several studies used over 10^6^ copies of sorted chromosomes (~0.1 pg for 100Mbp chromosome): 1.3 million human chromosome X [17] and 300 ng of mouse chromosome 17 [42] for SNP and CNV discovery based on Illumina GA; 80–620 ng of every sorting peak in a Chinese Hamster Ovary cell line for separated chromosome assembly based on Illumina HiSeq [43]. The DNA yields from a few hundreds of flow-sorted chromosomes and a few copies of microdissected chromosomes are far too low and require amplification prior to NGS.

**Fig. 3:**
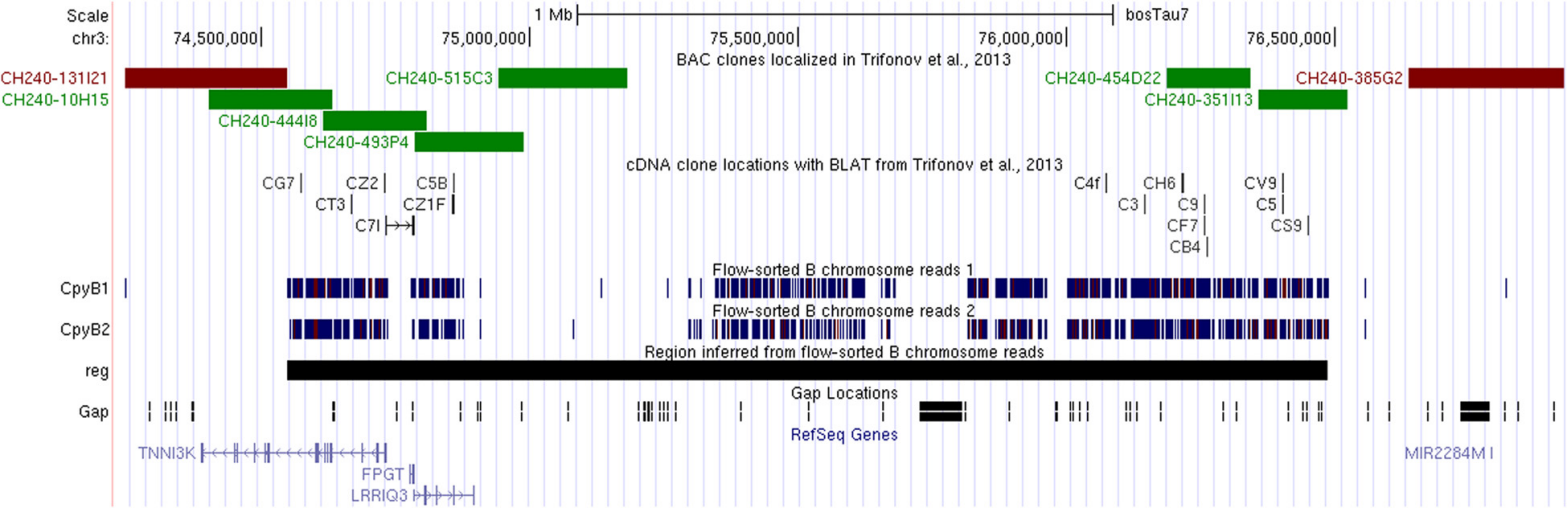
Region of BTA3 present on B chromosomes of Siberian roe deer. BAC clone localization (green – present, red – absent) and cDNA data taken from [7]. Two samples from one flow-sorting experiment were used to produce reads 1 and 2. Read coverage data omitted. Note two regions lacking reads – one is covered by BACs, the other corresponds to an assembly gap

In our study we utilized DOP-PCR, a method for Whole-Genome Amplification (WGA) based on PCR with semi-random primers [18] previously applied in high throughput sequencing of the microdissected whole human chromosome 1 and the short arm of chromosome 6 with only summary statistics of mapping calculated [22]. In our study we found that DOP-PCR derived sequencing data is suitable for characterization of both non-repetitive and repetitive DNA in sampled chromosomes, although biases in DNA composition resulted in uneven DOP-position distribution, e.g. false deletion observed in CPYB (Fig. 3) and distorted repeat content. On the other hand, due to the non-random 3`-end of DOP-primer, the same amplicons can be recovered from different samples. For example, in technical replicates CPYB1 and CPYB2 2,133 DOP-positions were repeatedly recovered from both libraries across all cattle chromosomes. 782 (totalling in 205,979 bp) of those were located in the detected target regions. This is over three quarters of DOP-positions in target regions for both samples, indicating saturation of sequencing results with the recoverable DOP-amplicons. In this respect, sequencing of DOP-PCR amplified DNA is similar to reduced representation sequencing approaches, such as RAD-seq [44], with a benefit of coping with individual chromosomes obtained by flow sorting or microdissection.

Detection of chromosomal rearrangement margins based on NGS of isolated chromosomes was one of the main objectives of our study. Previously, a similar problem was resolved for balanced translocations in human [45, 46]. In these studies, Illumina reads of MDA-amplified flow-sorted chromosomes with translocations were mapped to the reference human genome. Breakpoints were detected as changes in coverage at 1-bp resolution with a maximum likelihood-based algorithm. This approach was not applicable in our case, due to incomplete genome coverage by DOP-positions. Here we used the distances between positions covered by reads to locate the margins of the target regions: lower distances are characteristic for target regions present on chromosomes. Our classification script currently works only as a provisional tool to indicate putative target regions: visual inspection revealed that several positions can be erroneously included in or excluded from the target region. Finally, the exact margins of the rearrangements have to be then verified by means of PCR.

In general, our approach utilizing NGS of isolated chromosomes amplified with DOP-PCR is relatively low cost method, allowing for over 10 samples to be analysed in a single Illumina MiSeq run. Resolution of margins of the rearrangements by the read mapping method was about 3-5 kbp (mean distance between neighbouring DOP positions), the minimum size of detected regions was 20 kbp, and the only errors discovered thus far were false-negative deletions (marginal shrinking of the regions is also expected). Spectra of repeats were biased but comparable between samples. Recovery of multiple sequence variants allowed the analysis of their functional distributions (coding/non-coding, synonymous/non-nonsynonymous) and heterozygosity. Reproducibility of the genomic positions of DOP-amplicons enables further phylogenetic analysis.

### DNA content of B chromosomes in cervids

The *KIT* gene has been found on the B chromosomes of the grey brocket (this study) and two distinct species of canids [4–6]. *KIT* is a protooncogene encoding the receptor tyrosine kinase crucial for differentiation of haematopoietic, melanoblast and primordial germ cells. Mutations and amplification of this gene activate cancerogenesis in humans [47, 48] and lead to various pigmentation phenotypes in mammals (reviewed in [9, 49]). It is interesting that the control sample of mixed cattle chromosomes 23, 26, 28, and 29 indicates a translocation of a 480-kbp genomic region including *KIT* from BTA6 to one of these small autosomes. This translocation was previously described as linked with colour sidedness phenotype and the target chromosome was identified as BTA29 [24]. Sizes and exact coordinates of rearrangements involving *KIT* vary: 480 kbp region including the entire *KIT* gene in cattle translocation, 76 kbp region encompassing exons 1–20 of *KIT* in grey brocket Bs, 202 kbp including exons 2–21 of *KIT* in fox Bs.

*RET* represents another protooncogene found on B chromosomes of both grey brocket and a canid (Chinese raccoon dog). It encodes tyrosine-kinase and is frequently translocated in various cancers (reviewed in [50]). Among other cancer-related genes present on grey brocket Bs are: *PIK3CB* (phosphoinositide-3-kinase, catalytic, beta polypeptide) – an isoform of outer membrane kinase connected with DNA double-strand break repair and various types of cancer [51, 52]; *CCND2* (cyclin D2), controlling G1/S transition in cell cycle known to be translocated in mantle-cell lymphoma [53]; *OXSR1* (oxidative-stress responsive 1) – a serine/threonine kinase participating in stress response and cytoskeleton regulation, also was once identified as a weak candidate tumour suppressor [54].

According to the functional enrichment analysis, the most significant cluster (enrichment score 1.27) included 4 genes controlling embryonic development and cell differentiation: *ACVR2B*, *EOMES*, *GFI1*, and *KIT*. The second cluster (score 1.22) overlaps with the first one and includes kinases of proteins and amino acids (tyrosine – *KIT*, *RET*, *TXK*; serine/threonine – *ACVR2B*, *OXSR1*), nucleotides (*PIK3CB*) and carbohydrates (*XYLB* – xylulokinase homolog). A closely connected cluster of kinase positive regulation genes (score 0.91) includes three kinases (*ACVR2B*, *PIK3CB*, *KIT*), as well as *CCND2*. Most of these genes are located in separate regions, only *OXSR1*, *XYLB*, *ACVR2B* are found in a single 360-kbp region from cattle chromosome 22. If a connection between gene function and acquisition to B chromosome exists, it does not operate in the majority of cases: 5 out of 26 B-chromosomal regions bear cancer-related genes, 5 – kinases and their regulatory genes, other functional categories score even less regions.

Non-random content of B chromosomes fits well with the current knowledge of other types of genome instability on various time scales. For example, some genomic regions are more frequently amplified and translocated in human cancer than others [55]. Human small supernumerary marker chromosomes (sSMC) observed with an estimated population frequency of 1/2000 in most cases originate from a restricted number of genomic sites [1]. Reuse of some breakpoints in mammalian karyotype evolution and their enrichment with various features have been reported [56].

The B chromosomes of the Siberian roe deer incorporate only two regions less than 2-Mbp in size characterized by a high pseudogenisation and amplification of unique regions. A high degree of repeated sequences degradation further proves a degenerate nature of these genomic segments. Grey brocket deer Bs are completely different – there are 26 segments derived from different autosomal regions continuously ranging from a dozen kpb to over a Mbp in size. Pseudogenisation, internal amplification and repeat degradation were not significant. In contrast, a relatively new dispersal of LTR retrotransposons was detected.

Brocket deers and roe deers belong to different tribes of subfamily Capreolinae (Odocoileini and Capreolini, respectively) that diverged ca 7.4–7.8 Mya, early in Cervidae evolution [57]. An independent origin of Bs in these species coincides with the absence of Bs in other representatives of the subfamily. As an interesting exception, B chromosomes were found in 4 of ca. 10 *Mazama* species [10, 11]. Studies of the B chromosome genetic content in other *Mazama* lineages would test if they are of common or independent origins and thus specify their age as up to 5 million years (an estimated date of genus radiation [58]). As for the Siberian roe deer, the origin of B chromosomes could have occurred after its divergence from the European species [59], which has been dated by the mitochondrial control region phylogeny as approximately 2 Mya [60]. The resulting potential controversy (i.e., young but differentiated Bs in Siberian roe deer versus older but virtually intact Bs in *Mazama* species) awaits to be addressed by future studies.

## Conclusions

We have developed a method for the analysis of DOP-PCR amplified chromosome-specific DNA using Next-Generation Sequencing. It includes breakpoint mapping at a resolution of several thousand nucleotides, as well as information on sequence variation and repeat content. This approach is cost-effective: about 10 chromosomes can be analysed in a single Illumina MiSeq run. We propose a range of applications besides B chromosome research including chromosome attribution for scaffolds in genome assembly and characterization of evolutionary and clinical rearrangements. As an example, we a identified a 480-kbp translocation in cattle, which is associated with colour sidedness.

Using this method we have found that B chromosomes of the Siberian roe deer are more derived than the ones of the grey brocket deer, and they have undergone amplification and intensive pseudogenisation processes, while the B chromosomes of the grey brocket deer have retained a low copy number of autosomal genes on B chromosomes without obvious signs of the degeneration of both unique and repetitive sequences. These patterns may reflect differences in either the age or evolutionary fate of Bs in these two lineages.

## Methods

### Cell cultures and chromosome sorting

Fibroblast tissue cultures of the Siberian roe deer [38], grey brocket deer [61], cattle, and dog [62] were taken from the collections of the Department of Genome Diversity and Evolution, Institute for Molecular and Cell Biology, Novosibirsk and the Cambridge Resource Centre for Comparative Genomics, Department of Veterinary Medicine, Cambridge University, UK. The numbers of B chromosomes in cell cultures of the cervids used to chromosome flow sorting were: eight for the Siberian roe deer and three for the grey brocket deer.

Cell culturing, metaphase preparation and chromosome flow sorting were performed as previously described [7, 26]. In flow sorting experiments we collected about 300 copies of B chromosomes, which were well resolved and distinct from non-B chromosomal peaks.

### Generation of chromosome-specific DNA with DOP-PCR

Degenerate oligonucleotide-primed polymerase chain reaction (DOP-PCR) [18] with 6-MW primer (5'-CCGACTCGAGNNNNNNATGTGG-3') was used for the generation of chromosome-specific DNA from sorted chromosomes as previously described [63].

### BAC clones

Cattle BAC clones from the CHORI-240 library listed in Additional file 1: Table S1 were used. Coordinates in the cattle genome (Baylor Btau_4.6.1 or bosTau7 in UCSC Genome Browser notation) were obtained from the NCBI Clone database or estimated from alignment of insert draft sequence to the genome with BLAST. Canine BAC-clone from RPC181 canine 8.1-fold BAC library containing *KIT* gene was previously characterized [4].

### Fluorescence *in situ* hybridization

Probes for fluorescence in situ hybridization (FISH) were labelled directly by DOP-PCR (for chromosome-specific DNA) or by using a nick translation kit (Invitrogen, UK) for bovine BACs. FISH was performed using a standard protocol [23, 62].

### cDNA library construction and sequencing

All procedures for grey brocket deer cDNA library construction, enrichment cloning and sequencing were performed as described [7]. Briefly, total RNA was extracted from grey brocket deer fibroblast tissue cell culture. A total cDNA library was constructed followed by enrichment for transcripts homologous to B chromosomes by selection of hybrids with affinity capture. Individual cDNA fragments were cloned and Sanger sequenced.

### Next Generation Sequencing

Sequencing of DOP-PCR chromosome-specific DNA was prepared using the Nextera DNA Library Preparation kit, which includes random fragmentation ofBEDTools: a flexible suite of utilities for comparing genomic features template DNA. 8 barcoded samples were sequenced in a single Illumina MiSeq run with read length equal to 150 bp and thus obtained 0.71–1.03 million reads per sample (Table 1). Sequencing data are freely available from NCBI read archive [SRA:PRJNA285957].

### QC, alignment to reference genomes and elimination of human contaminant reads

Sequencing adapters and DOP-primer were trimmed using cutadapt [64]. Three reference genomes were used: cattle (Baylor Btau_4.6.1 or bosTau7 in UCSC Genome Browser notation), dog (Broad CanFam_3.1 or canFam3) and human (GRCh37 or hg19). Reads were aligned to human (contamination) and either cattle or dog (target) genomes (see Table 1) using bowtie2 [65] in paired-end mode with “--local” option enabled for optimization of divergent sequence alignments. Only highly significant and unique alignments to the target genome with MAPQ > 20 were left. After that we removed the reads that aligned better to contamination than to the target genome based on MAPQ scores.

### Detection of target regions

BAM files with mapped reads were converted to BED files with positions and coverage information using BEDtools [66] “bamtobed -i” and “merge -n -i” commands. The genome was divided into regions with differing mean distance between positions with a custom R script region_dnacopy.R. First, for each chromosome distances are calculated between every DOP-position and the position on its left (PD) (for the first position in the chromosome, distance to 0-coordinate is taken). Then, changes of mean PD along the chromosomes are located. This step relies on the DNAcopy R package which uses circular binary segmentation and was initially designed for the detection of copy number variations from microarray genotyping data [67]. For this analysis, we found it useful to remove outlier values: big PD values corresponding to genomic regions without any positions, which are most likely unmappable (gaps or repeats); small values representing non-overlapping reads from one amplicon. Lastly, the script plots the results (Additional file 2) and outputs a table with chromosomes divided into regions and several statistics for each region. Regions with lowest mean PD are candidates for being target (i.e. present on the sampled chromosome).

Within the target regions, pairwise distances between positions were highly unevenly distributed with numerous long gaps (e.g., Fig. 2). We propose several explanations for this: 1) deletions or rapid sequence divergence occurred after duplication 2) reads cannot be mapped significantly to duplicated and repetitive genome regions, for example, human contamination reads are more or less evenly distributed throughout the genome, but no reads are mapped to short arms of chromosomes 21 and 22, where clusters of ribosomal genes are located; 3) reads cannot be mapped to gaps, including conventional 3 Mbp gaps at centromeres; 4) genomic sequence biases resulting in lack of ATGTGG motif and 5) PCR amplification biases. We accounted for factor 3) as gap positions throughout the genome are known. Combination of factors 2) and 4) can theoretically be assessed by mapping of putative DOP-positions flanked by the ATGTGG hexamer, but this method is prone to nucleotide substitutions, especially for cross-species alignment. We accounted for stochastic DOP-fragment retention by independent generation of two samples from one sorting experiment of Siberian roe deer B chromosomes. The observed level of agreement was high enough to make this factor negligible. BAC clone localization in both Siberian roe deer [7] and grey brocket deer (Additional file 1: Table S1) were in agreement with sequencing-derived regions, even partial overlap of the BAC and the region was sufficient to generate a hybridization signal. The only exception occurred in Siberian roe deer, where the BAC clone signals were positive for a region not covered by reads in both libraries (Fig. 2, clone CH240-515C3). As BAC-clone localization evidence is direct, we should recognize the extent of reproducible biases in DOP-PCR amplified DNA composition.

### Functional gene enrichment analysis

Lists of complete and complete + partial genes present on B chromosomes of the grey brocket deer were analysed for GO function enrichment with DAVID [68, 69]. As a background we used the cattle gene list from the database.

### Sequence variation analysis

For chromosome-specific read alignments sequence variants were called using GATK HaplotypeCaller [70] with default options. Variant manipulation, comparison and statistics calculation were made with appropriate GATK instruments for all and heterozygous only variants. Variant density was calculated by dividing the total size of positions covered by reads in target regions by the number of variants called. Variants were annotated using Variant Annotation Integrator at UCSC genome browser [71] based on cattle RefSeq genes. Size of gene features (intergenic, intron, 5` UTR, 3` UTR, coding sequence) covered by reads were calculated by intersection with the appropriate fraction of cattle RefSeq genes (track used for variant annotation).

The bovine dbSNP 138 dataset [30] was downloaded from UCSC genome browser ftp. The dataset was converted to GATK-compatible format. About 40 thousand of the 22.3 million variants were filtered out due to malformations.

To obtain sequence variants of the European roe deer, its genomic contigs [31] were aligned to bosTau7 genome using BWA-MEM [72]. All variants present in uniquely mapped contigs were called with GATK UnifiedGenotyper with options “-glm BOTH -minIndelCnt 1 -indelGOP 80 -stand_call_conf 0 -stand_emit_conf 0” in order to output all variants present in every contig. We called all variants for regions with >1 contig aligned simultaneously (i.e. putative duplications in European roe deer versus cattle), thus the total number of variants might be inflated.

### Repetitive DNA content

We implemented a *de novo* repeat characterization approach utilizing clustering and annotation of reads with RepeatExplorer [35, 36]. Trimmed but not filtered reads of length >19 bp were submitted to RepeatExplorer clustering algorithm on Galaxy-based server (http://www.repeatexplorer.org/). Read similarities over 55 % of the read length were interpreted as edges connecting the similar reads (nodes). Clusters of frequently connected reads (each including >0.01 % of initial reads) were annotated with mammalian RepeatMasker database (http://www.repeatmasker.org).

## Additional files

Additional file 1:Table S1. Bovine BAC clone localization on grey brocket deer chromosomes. **Table S2.** Repeat family composition. **Table S3.** Major repeat clusters revealed in chromosome-specific DNA with RepeatExplorer. **Figure S1.** Insert length distribution for chromosome-specific DNA containing bovine chromosomes 23, 26, 28 and 29) inferred from paired-end read mapping to cattle genome. **Figure S2.** Fluorescence *in situ* hybridization of chromosome-specific DNA samples containing mixed bovine chromosomes 23, 26, 28 and 29 (red - CY3) and chromosome 10 (green - FITC) to cattle metaphase and corresponding DAPI staining. **Figure S4.** Fluorescence *in situ* hybridization of bovine BAC clone CH240-472E2 to grey brocket deer metaphase and corresponding DAPI staining. (DOC 633 kb) Additional file 2: Distance between positions plots. Plots are presented for all chromosomes of reference genomes (canFam3 for CFA12, bosTau7 for BTAMix, CPYB1, CPYB2, and MGOB). Axes: X – ln(distance between consecutive DOP-positions, bp), Y – cumulative chromosome length, bp. Black dots – individual distances, red lines – mean values. (PDF 958 kb)
