# Supplementary material for: Contrasting origin of B chromosomes in two cervids (Siberian roe deer and grey brocket deer) unravelled by chromosome-specific DNA sequencing
Source: BMC Genomics. 2016 Aug 11;17:618. doi: 10.1186/s12864-016-2933-6 (PMC4982142; doi:10.1186/s12864-016-2933-6)

Table S1. **Bovine BAC clone localization on grey brocket deer chromosomes.** FISH signal strength: '-' - none, '+' - medium, '++' - high.

| BAC clone | Genome coordinates | Signal |
| --- | --- | --- |
| CH240-29P21 | BTA7:22,018,658-22,199,463 | - |
| CH240-424I20 | BTA7:21,030,383-21,196,139 | - |
| CH240-473J3 | BTA10:22,462,589-22,651,807 | - |
| CH240-472E2 | BTA10:29,027,589-29,240,194 | + |
| CH240-449J24 | BTA10:29,183,189-29,358,021 | - |
| CH240-374L11 | BTA29:49,496,901-49,663,736 | ++ |
| CH240-244F2 | BTA29:49,586,750-49,749,643 | ++ |
| CH240-235P20 | BTA29:50,039,460-50,203,667 | ++ |
| CH240-39K2 | BTA29:50,184,810-50,412,902 | ++ |

Table S2. **Repeat family composition.** Percentage of genome sequence is given for bovine chromosomes 23, 26, 28, 29 according to RepeatMasker annotation of bosTau7 genome assembly. For sequencing libraries, percentage of annotated clustered reads versus all analyzed reads is given. Two numbers for Siberian roe deer B chromosomes (CPYB) correspond to independent library construction experiments. Only repeat families with frequency over 0.1% in at least one dataset are included

|  | bosTau7_chr | BTAMix | CPYB | MGOB |
| --- | --- | --- | --- | --- |
| **Total** | **43.90** | **42.73** | **77.76/79.45** | **63.23** |
| Unknown | 0.10 | 16.62 | 52.33/47.70 | 28.90 |
| Satellite.centr | 0.11 | 7.81 | 5.77/8.84 | 4.41 |
| Satellite | - | 0.10 | 4.84/8.11 | 1.49 |
| Simple_repeat | 0.50 | 0.23 | 1.32/2.06 | 0.76 |
| Low_complexity | 0.41 | 0.03 | 0.05/0.07 | 0.08 |
| LINE.L1 | 12.12 | 4.98 | 6.55/5.36 | 9.67 |
| LINE.RTE-BovB | 5.69 | 1.76 | 2.17/2.10 | 1.35 |
| LINE.L2 | 2.29 | 0.08 | 0.33/0.26 | 0.45 |
| LINE.CR1 | 0.31 | 0.05 | -/- | 0.01 |
| LINE-RT (RTE-X) | 0.08 | - | -/- | 0.10 |
| SINE.BovA | 6.46 | 2.24 | -/- | 6.62 |
| SINE.RTE-BovB | 5.46 | 7.48 | 1.60/2.12 | 3.49 |
| SINE.MIR | 2.50 | 0.24 | 0.20/0.18 | 0.42 |
| SINE.tRNA-Glu | 1.59 | 0.39 | 0.28/0.36 | 1.52 |
| SINE.Alu | - | 0.06 | 0.03/0.02 | 0.75 |
| LTR.ERVL-MaLR | 1.39 | 0.06 | 0.12/0.09 | 0.29 |
| LTR.ERV1 | 1.27 | 0.13 | 0.91/0.78 | 0.90 |
| LTR.ERVL | 0.90 | 0.05 | 0.06/0.04 | 0.10 |
| LTR.ERVK | 0.54 | 0.26 | 0.20/0.13 | 1.52 |
| DNA.hAT-Charlie | 1.13 | 0.05 | 0.07/0.09 | 0.23 |
| DNA.TcMar-Tigger | 0.46 | 0.07 | 0.02/0.01 | 0.02 |
| DNA.hAT-Tip100 | 0.22 | - | -/- | 0.00 |
| DNA.hAT-Blackjack | 0.10 | 0.02 | -/- | 0.01 |

Table S3. **Major repeat clusters revealed in chromosome-specific DNA with RepeatExplorer.** Clusters with >1% reads are included for each library. Only annotations for >10% reads in clusters are listed.

A) Cattle chromosomes 23, 26, 28, 29

| # | Read % | Annotation |
| --- | --- | --- |
| CL1 | 5.12 | Satellite.centr (98.4%) |
| CL2 | 4.35 | SINE.RTE.BovB (95.5%) |
| CL3 | 3.39 | SINE.RTE.BovB (87.8%) |
| CL4 | 2.67 | SINE.BovA (80.7%) |
| CL5 | 2.08 | Satellite.centr (98.7%) |
| CL6 | 1.36 | LINE.L1 (75.8%) |
| CL7 | 1.32 | Unknown |

B) Siberian roe deer B chromosomes. Sample 1.

| # | Read % | Annotation |
| --- | --- | --- |
| CL1 | 11.00 | Unknown |
| CL2 | 9.06 | Unknown |
| CL3 | 5.13 | Unknown |
| CL4 | 4.83 | Satellite.centr (65.5%) Satellite (19.6%) |
| CL5 | 4.31 | Satellite (66%) Simple_repeat (21.8%) |
| CL6 | 4.31 | Unknown |
| CL7 | 3.86 | Simple_repeat (8.63%) |
| CL8 | 3.08 | Unknown |
| CL9 | 2.58 | SINE.RTE.BovB (60.4%) LINE.RTE.BovB (18.2%) |
| CL10 | 2.39 | Satellite.centr (77.1%) Satellite (11.8%) |
| CL11 | 2.36 | LINE.L1 (92.3%) |
| CL12 | 1.94 | LINE.RTE.BovB (64.8%) |
| CL13 | 1.55 | LINE.L1 (98%) |
| CL14 | 1.34 | Satellite.centr (57.4%) Satellite (22.8%) |
| CL15 | 1.31 | SINE.BovA (36%) |
| CL16 | 1.17 | Unknown |

C) Grey brocket deer B chromosomes

| # | Read % | Annotation |
| --- | --- | --- |
| CL1 | 4.19 | SINE.BovA (77.5%) |
| CL2 | 3.80 | SINE.RTE.BovB (77.2%) |
| CL3 | 2.51 | Satellite.centr (95.5%) |
| CL4 | 2.01 | LINE.L1 (65.8%) Satellite (30.1%) |
| CL5 | 1.36 | LTR.ERVK (100%) |
| CL6 | 1.26 | LINE.L1 (57%) SINE.tRNA.Glu (12%) |
| CL7 | 1.23 | LINE.L1 (97.2%) |
| CL8 | 1.21 | SINE.BovA (47.4%) |
| CL9 | 1.16 | LINE.L1 (31.6%) |
| CL10 | 1.12 | Satellite.centr (67.9%) Satellite (38.7%) |

Figure S1. **Insert length distribution for chromosome-specific DNA containing bovine chromosomes 23, 26, 28 and 29) inferred from paired-end read mapping to cattle genome.** Note two peaks corresponding to virtually intact and fragmented DOP-PCR amplicons.


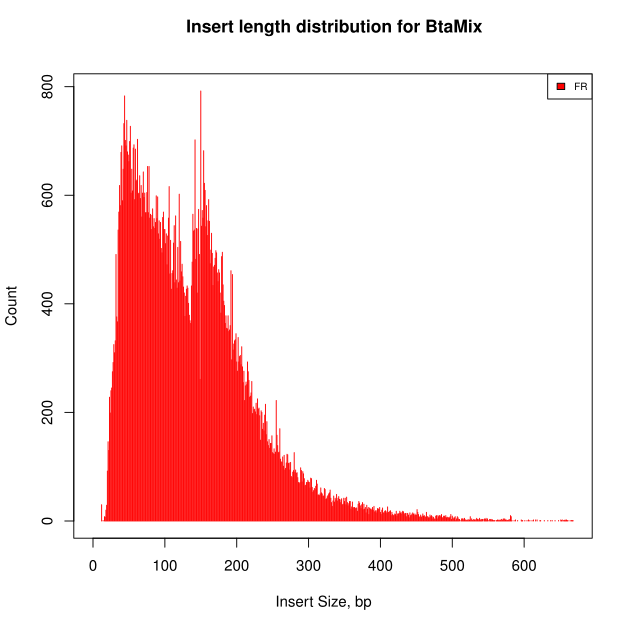


Figure S2. **Fluorescence *in situ* hybridization of chromosome-specific DNA samples containing mixed bovine chromosomes 23, 26, 28 and 29 (red - CY3) and chromosome 10 (green - FITC) to cattle metaphase and corresponding DAPI staining.**


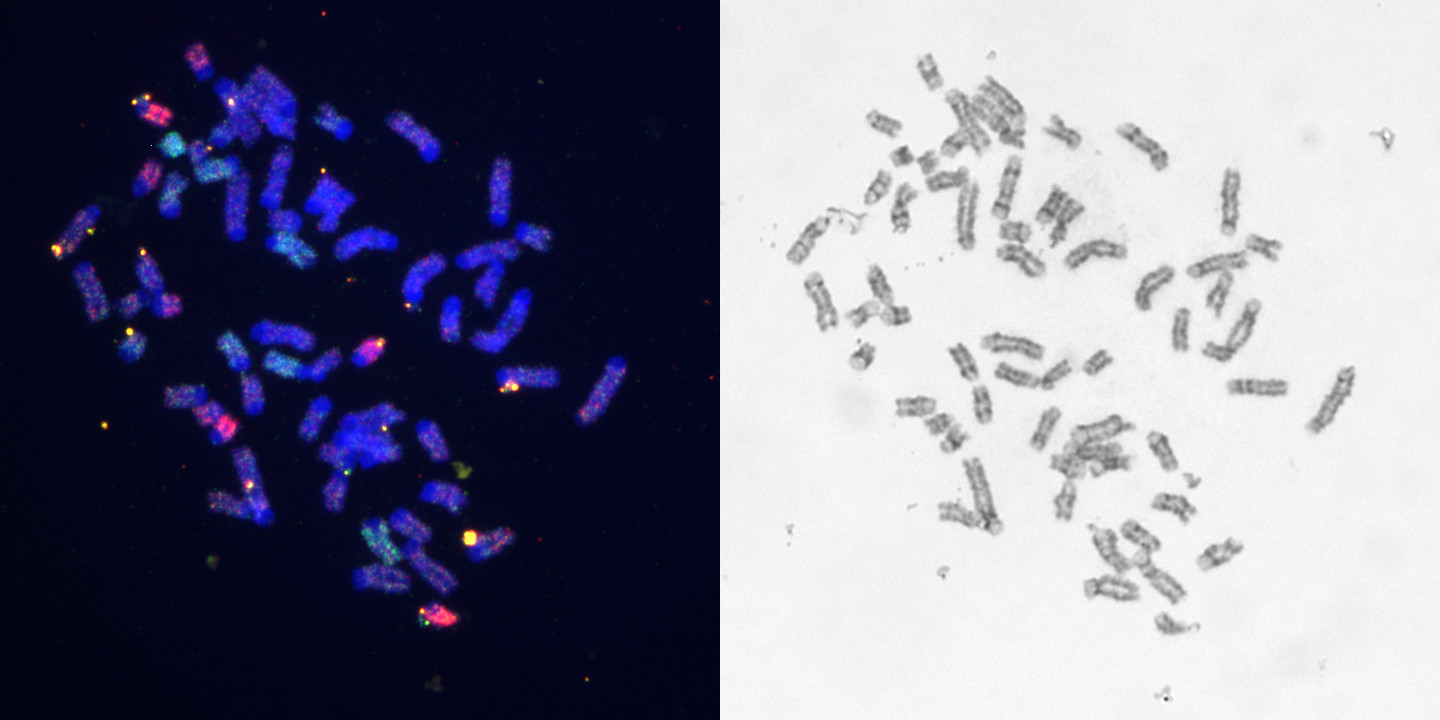


Figure S3. **Fluorescence *in situ* hybridization of grey brocket deer B chromosome-specific DNA to grey brocket deer metaphase and DAPI staining.** B chromosomes are indicated with arrows.


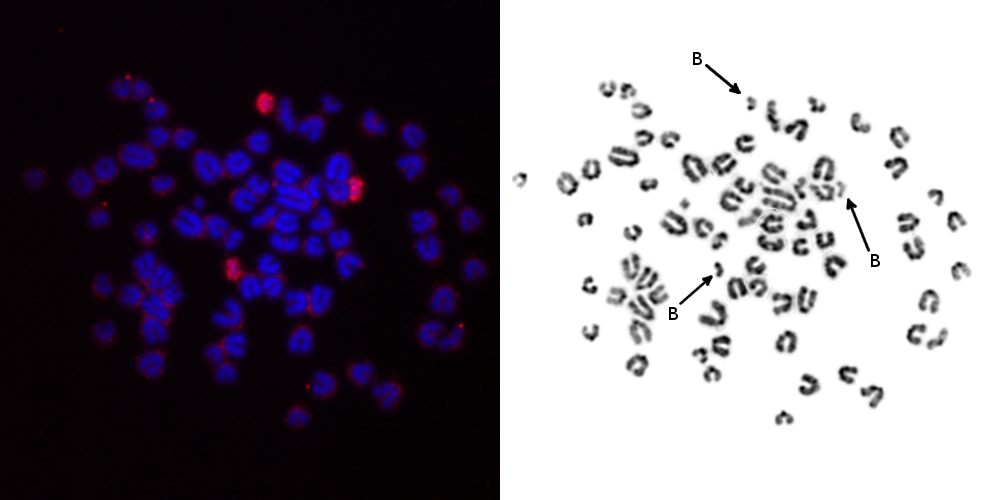


Figure S4. **Fluorescence *in situ* hybridization of bovine BAC clone CH240-472E2 to grey brocket deer metaphase and corresponding DAPI staining.** B chromosomes are indicated with arrows.


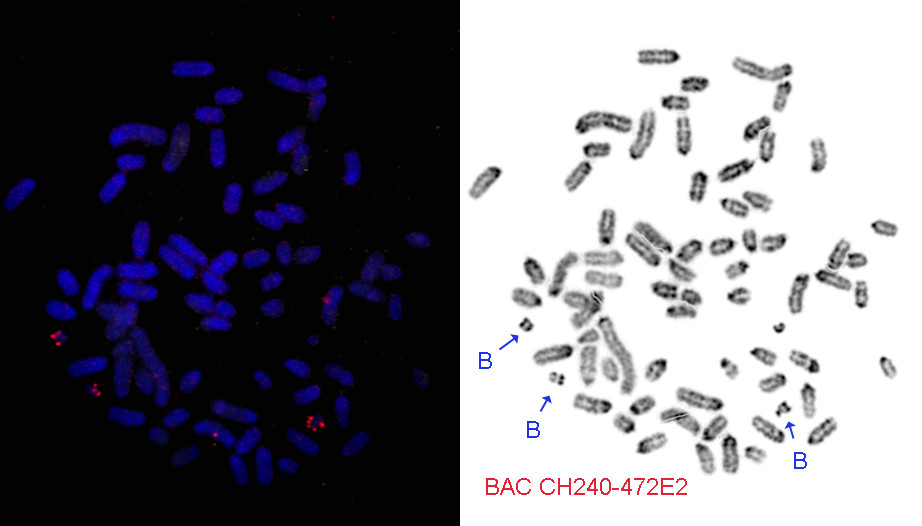

Supplement: Additional file 1:Table S1. — Bovine BAC clone localization on grey brocket deer chromosomes. Table S2. Repeat family composition. Table S3. Major repeat clusters revealed in chromosome-specific DNA with RepeatExplorer. Figure S1. Insert length distribution for chromosome-specific DNA containing bovine chromosomes 23, 26, 28 and 29) inferred from paired-end read mapping to cattle genome. Figure S2. Fluorescence in situ hybridization of chromosome-specific DNA samples containing mixed bovine chromosomes 23, 26, 28 and 29 (red - CY3) and chromosome 10 (green - FITC) to cattle metaphase and corresponding DAPI staining. Figure S4. Fluorescence in situ hybridization of bovine BAC clone CH240-472E2 to grey brocket deer metaphase and corresponding DAPI staining. (DOC 633 kb) [file 12864_2016_2933_MOESM1_ESM.doc]
