# Supplementary figures and images for: Contrasting origin of B chromosomes in two cervids (Siberian roe deer and grey brocket deer) unravelled by chromosome-specific DNA sequencing
Source: BMC Genomics. 2016 Aug 11;17:618. doi: 10.1186/s12864-016-2933-6 (PMC4982142; doi:10.1186/s12864-016-2933-6)

CFA12

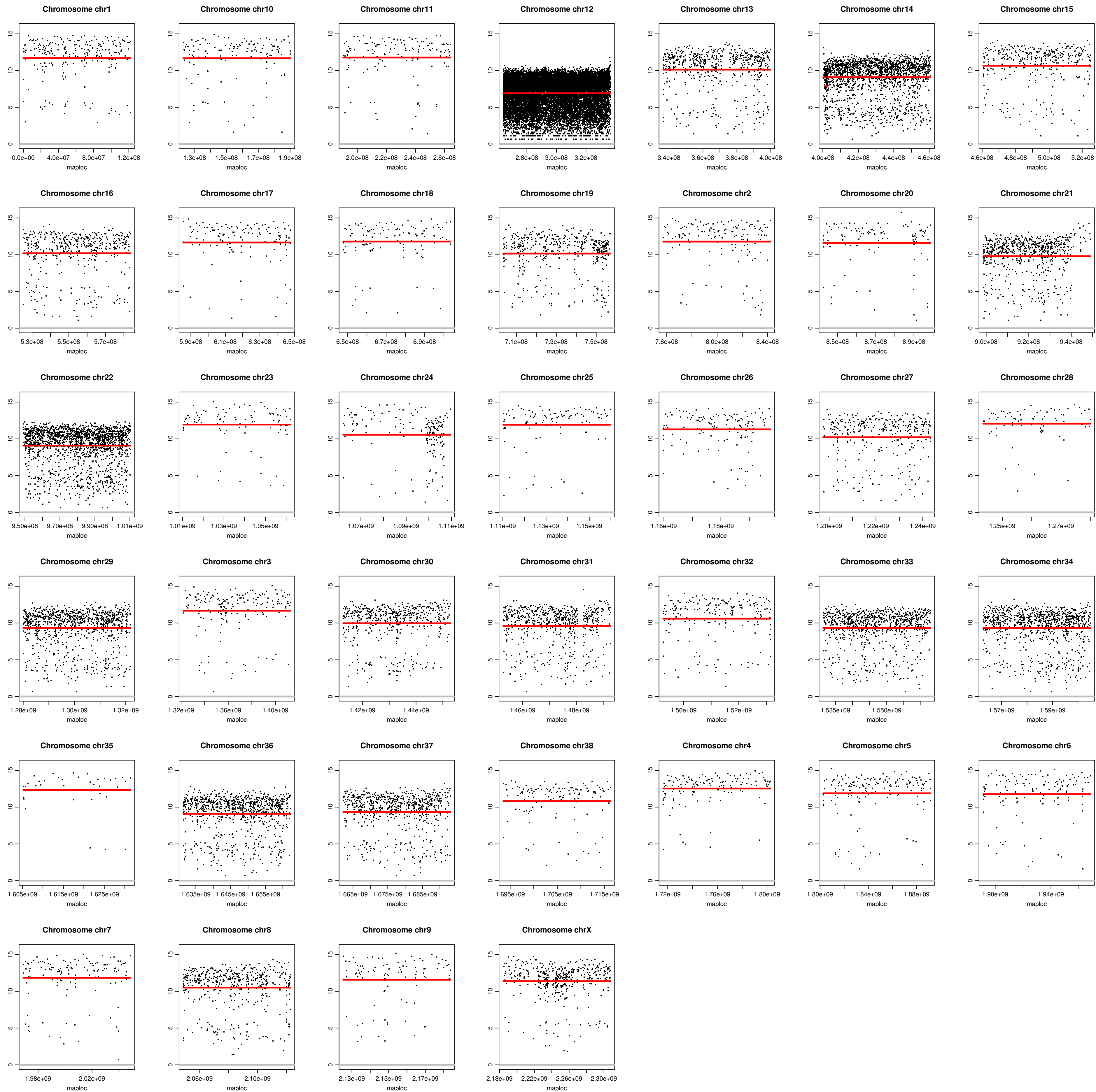

## BTAMix

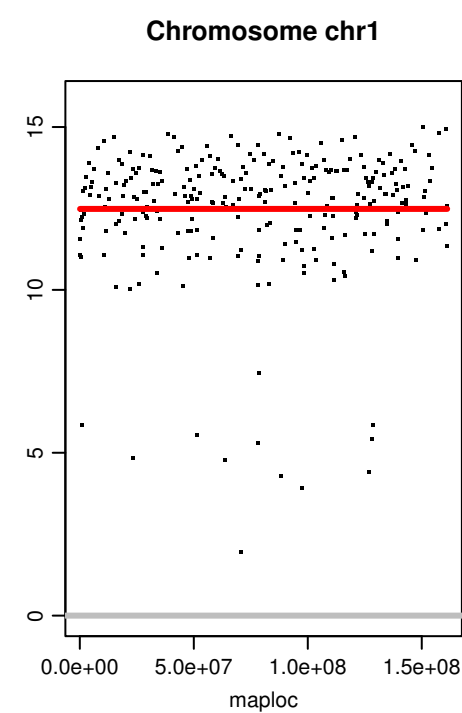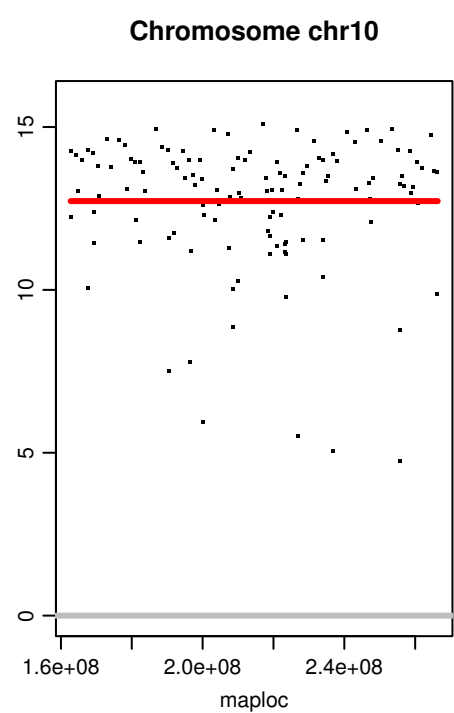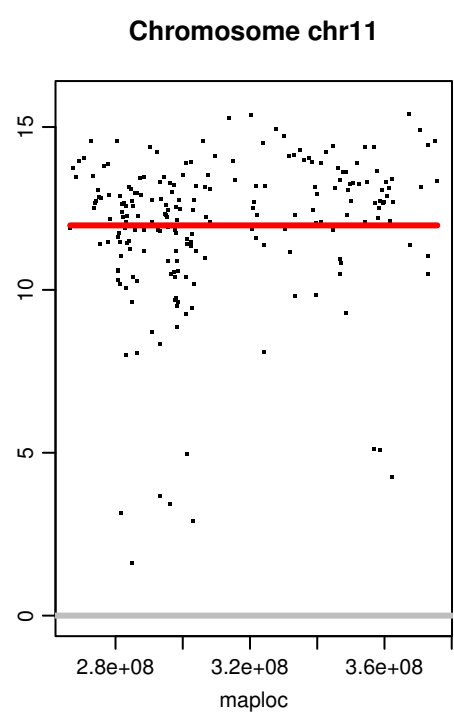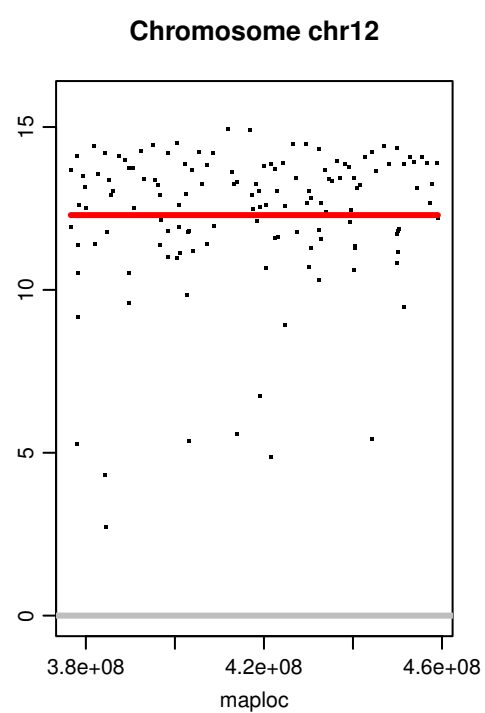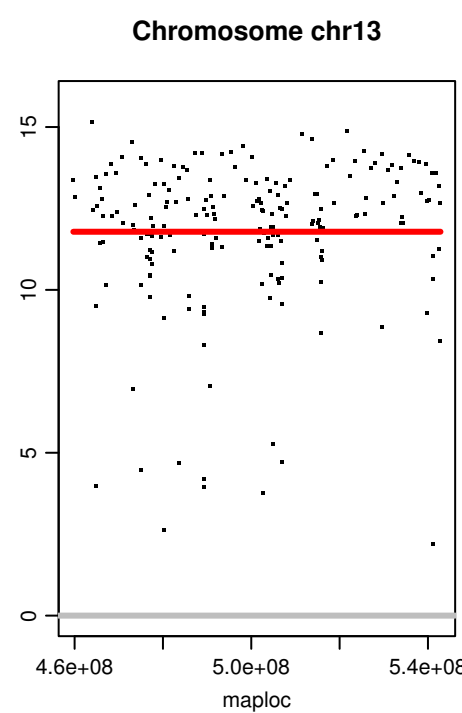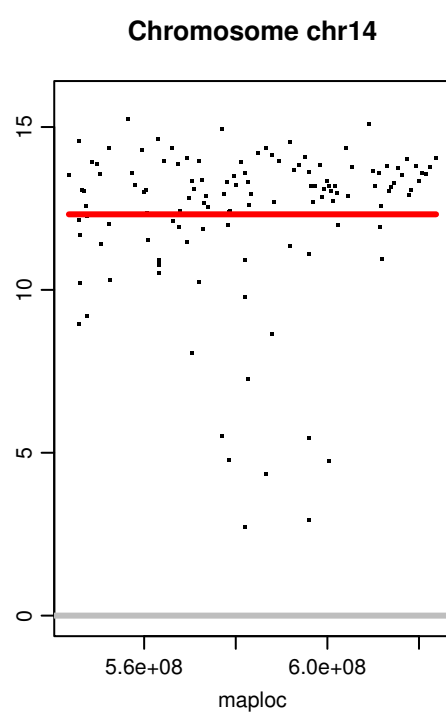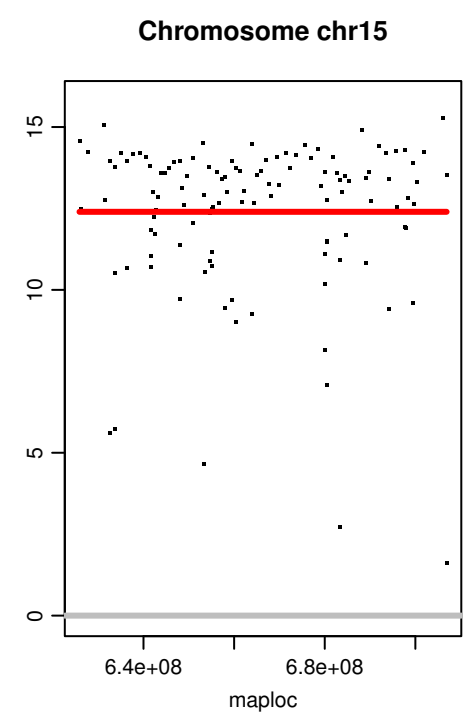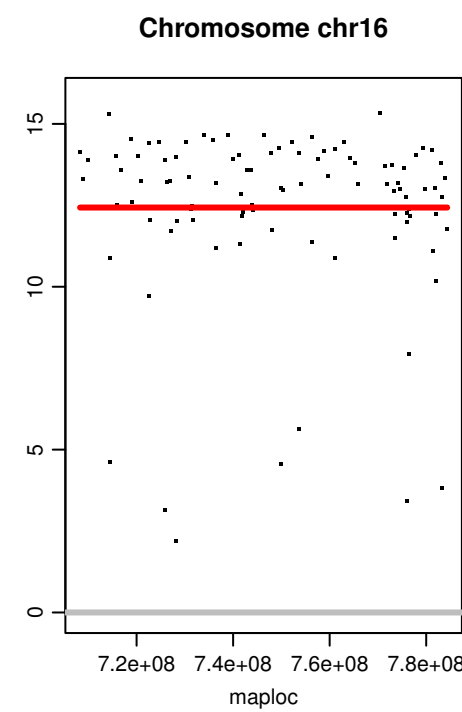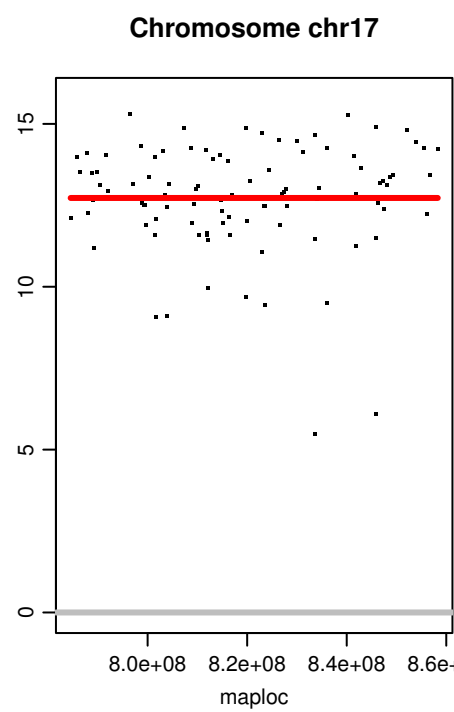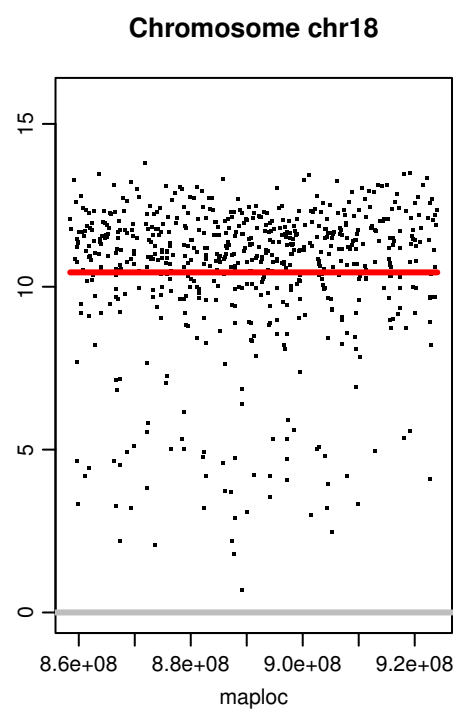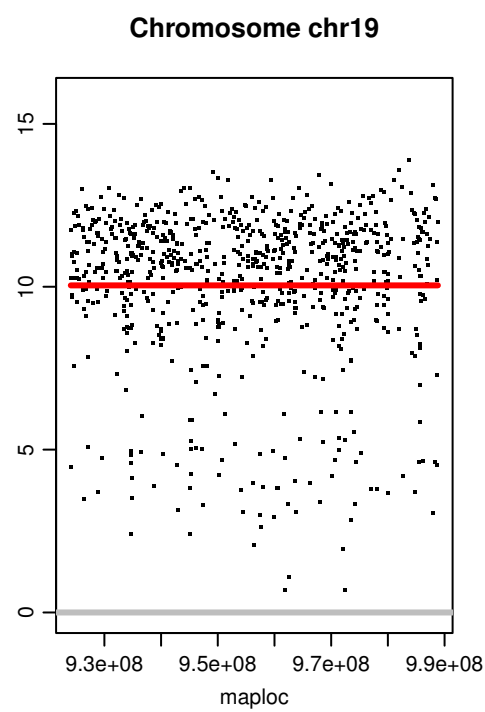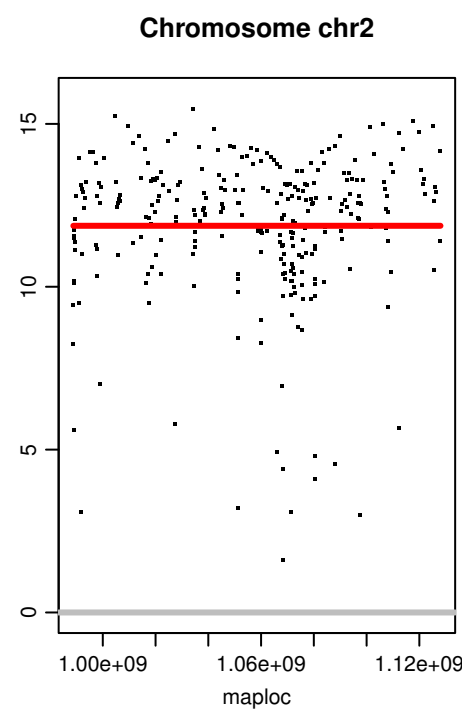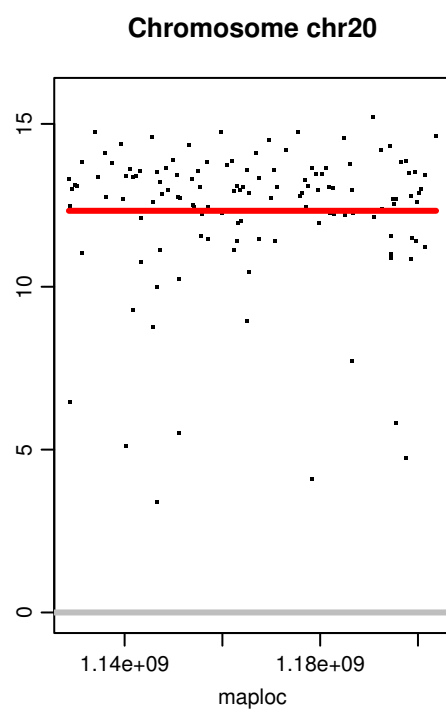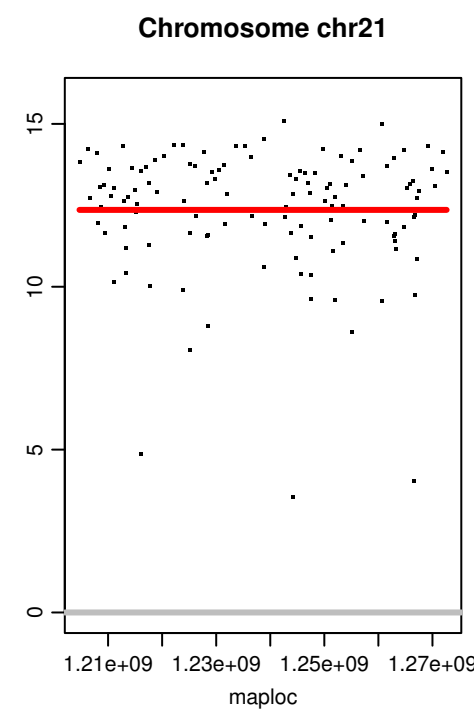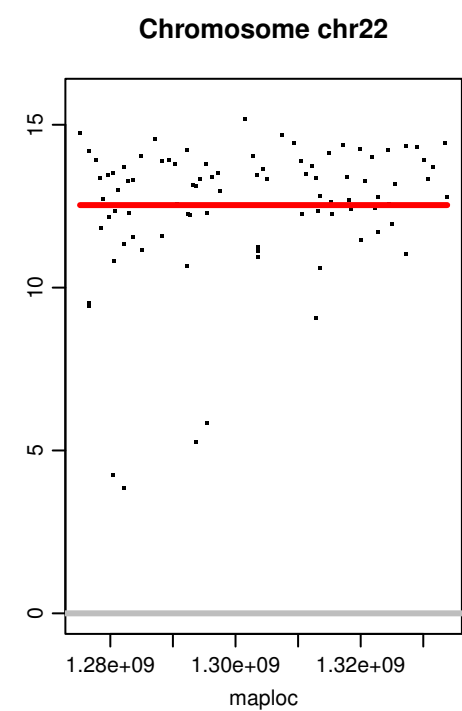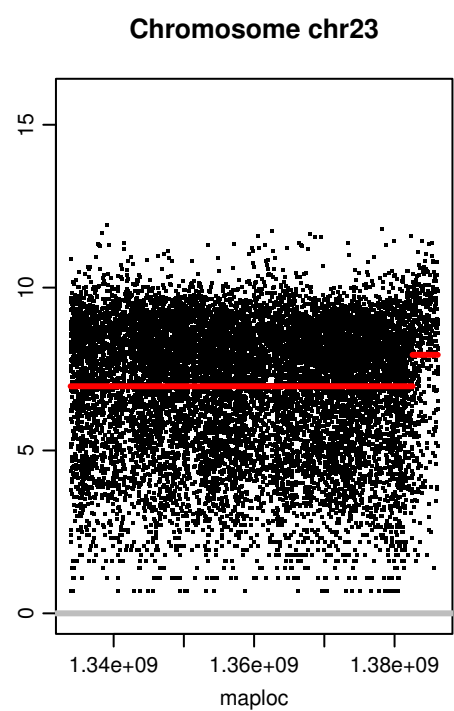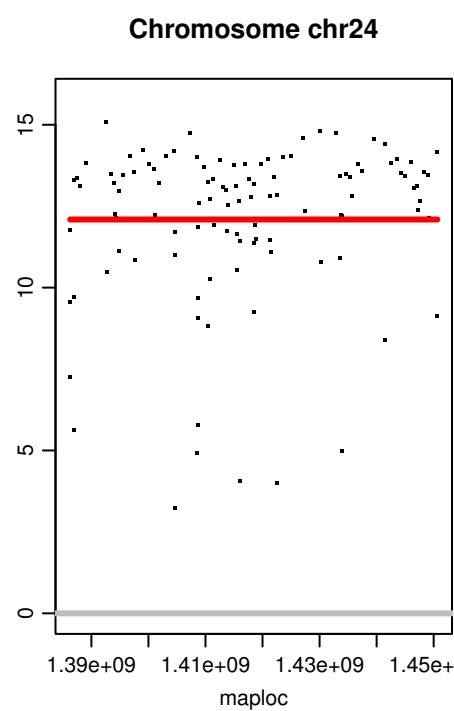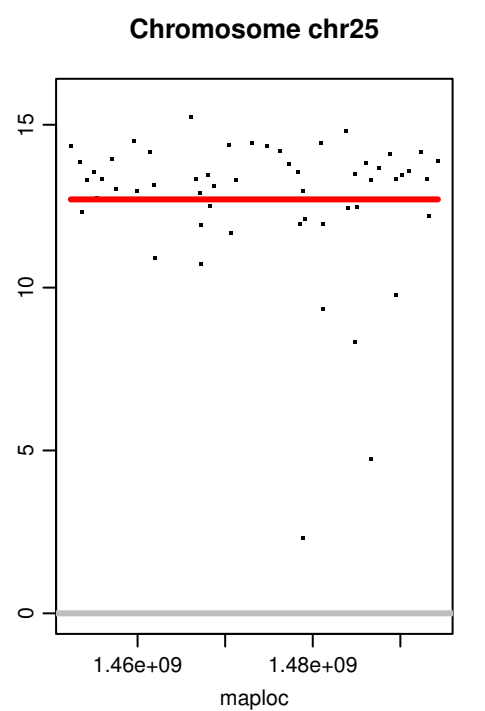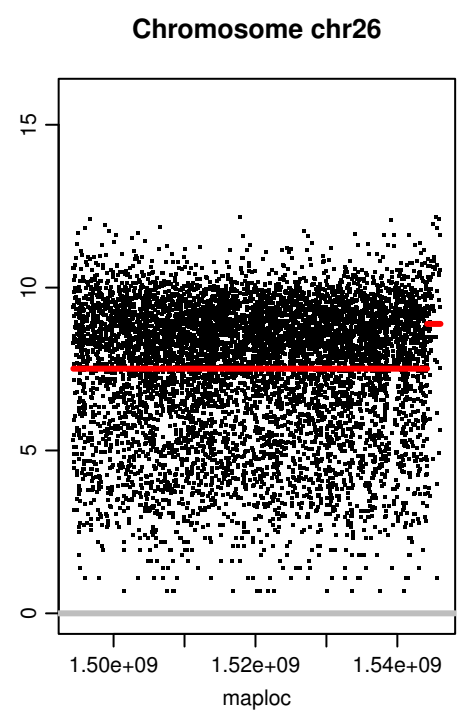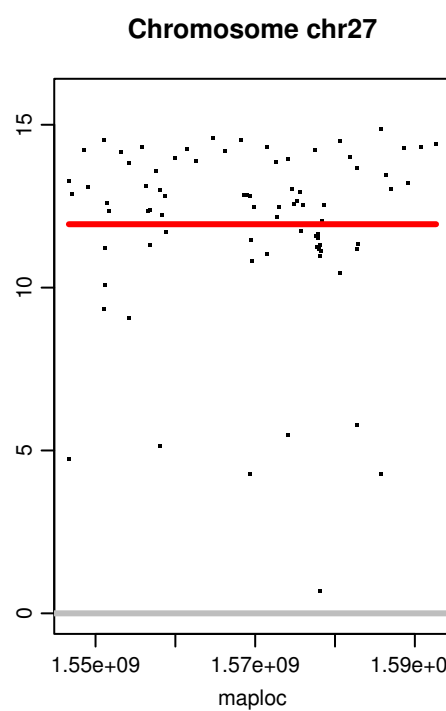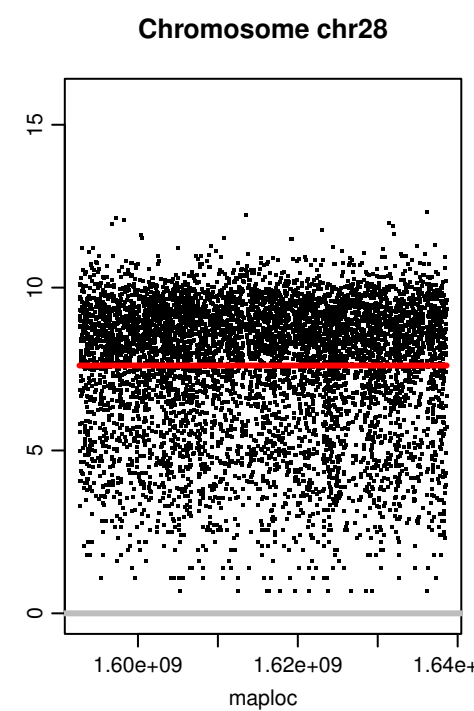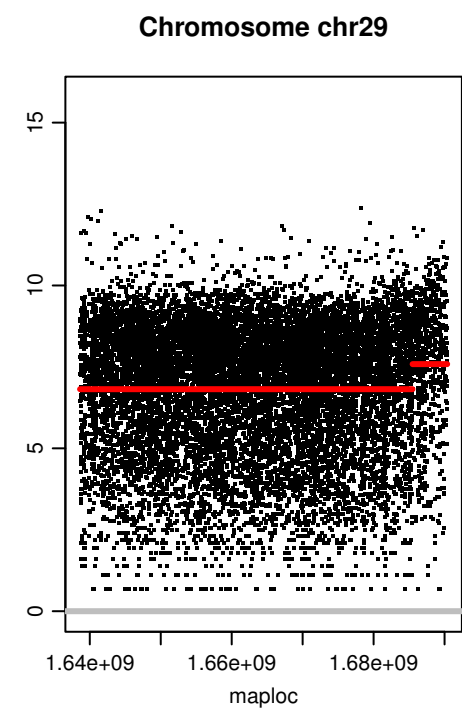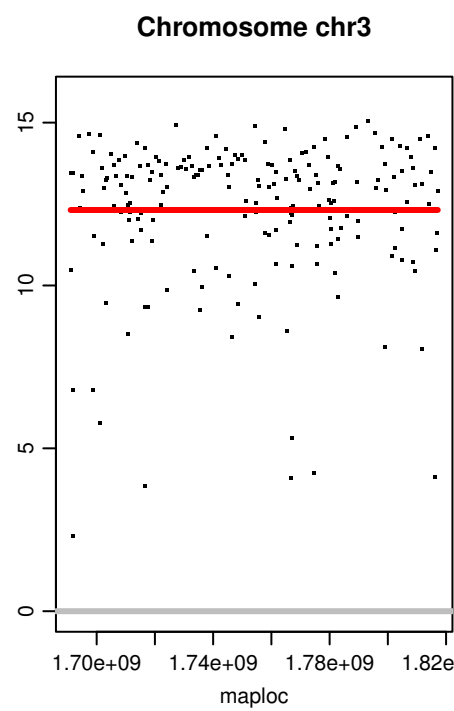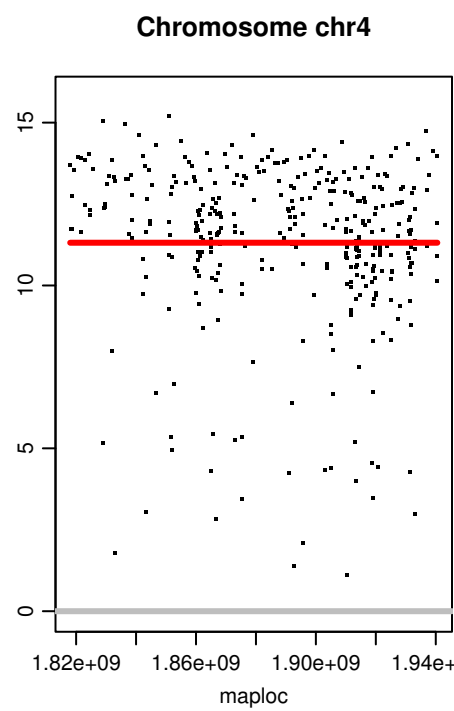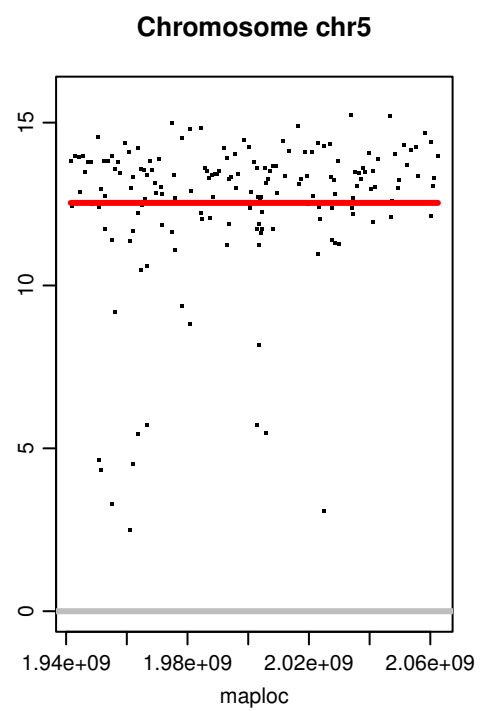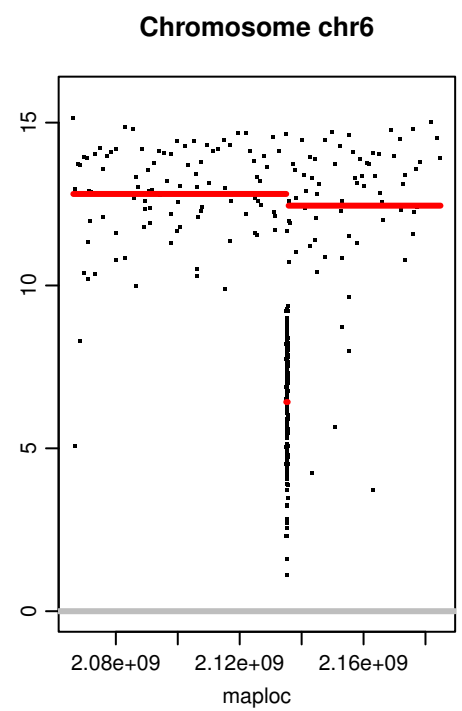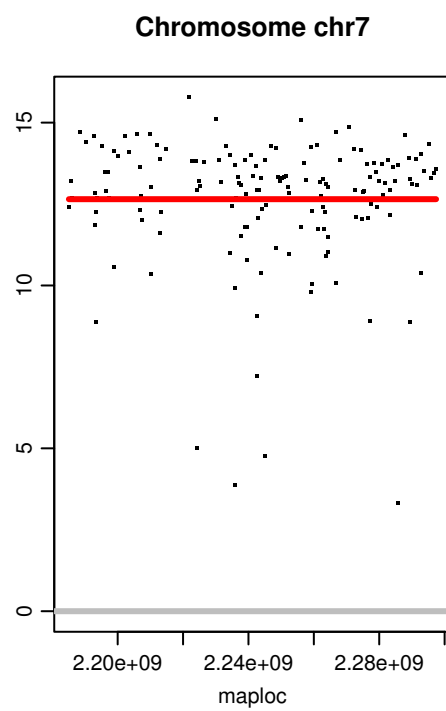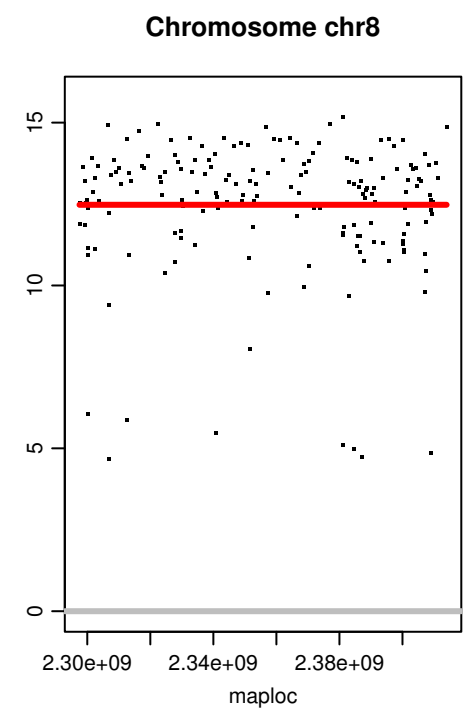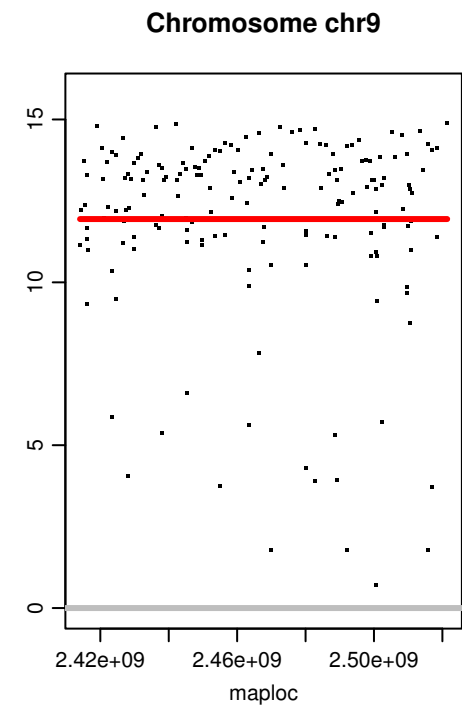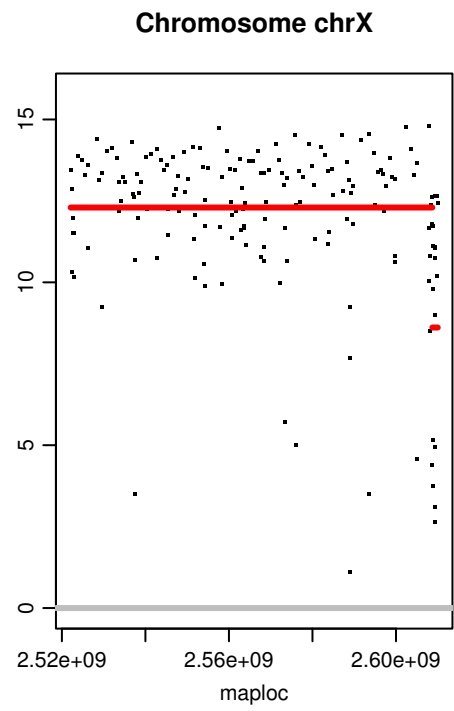

CPYB1

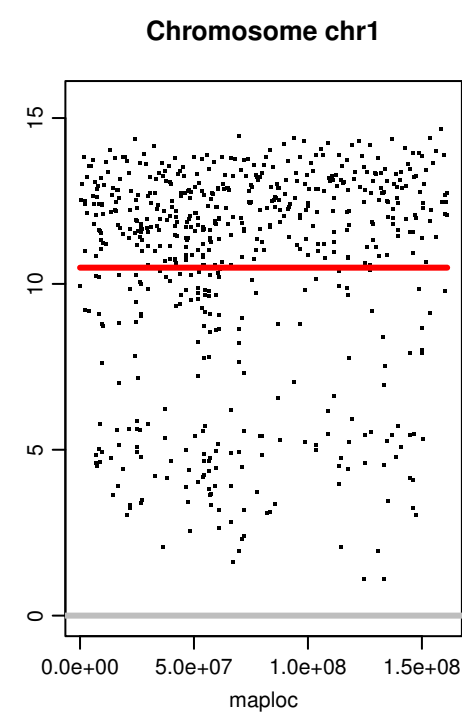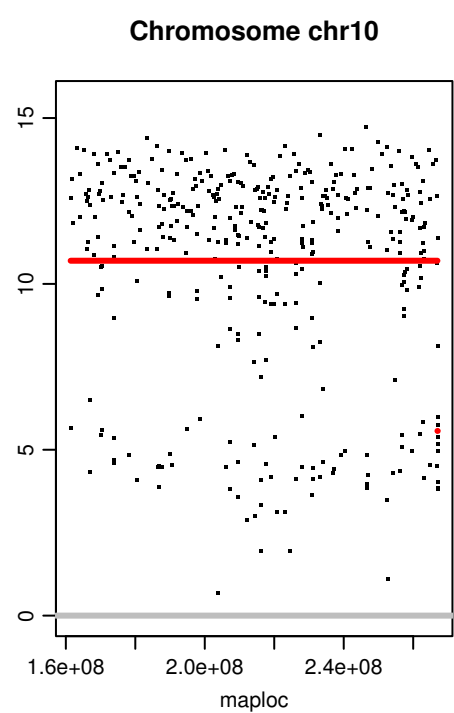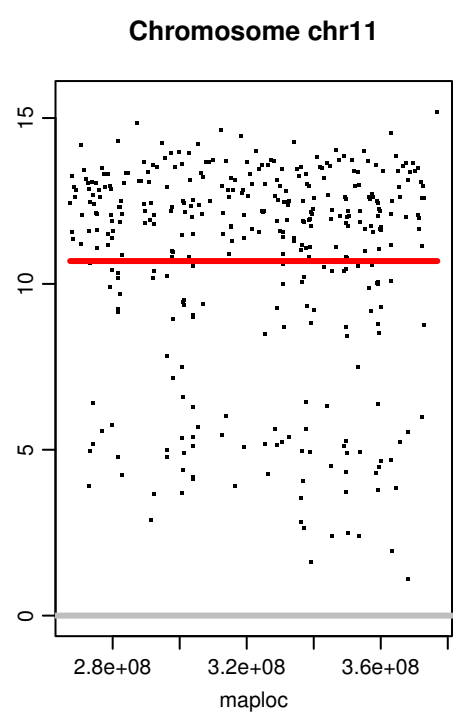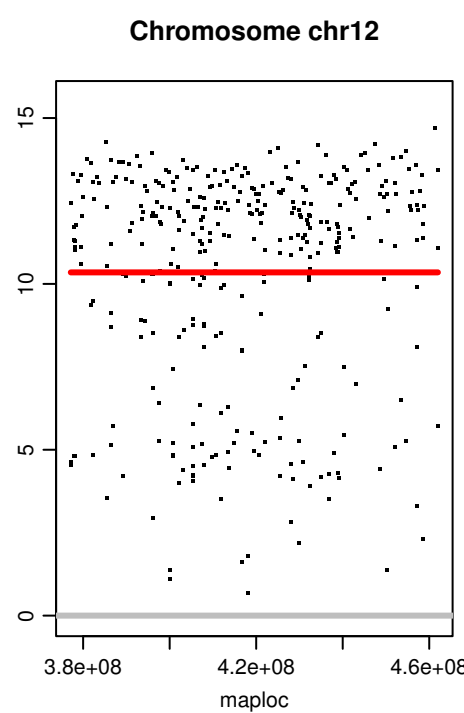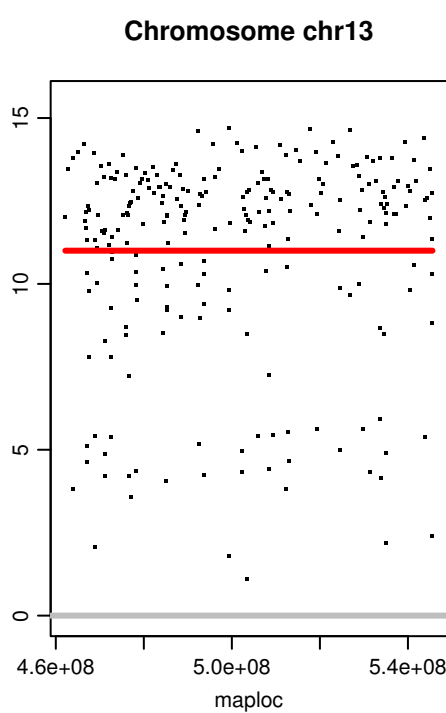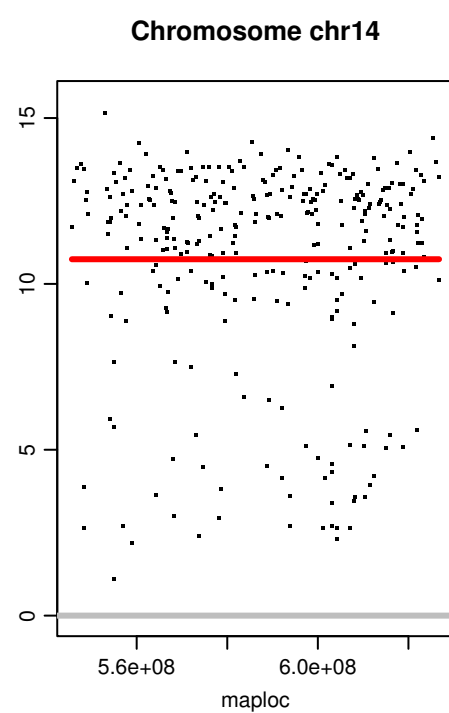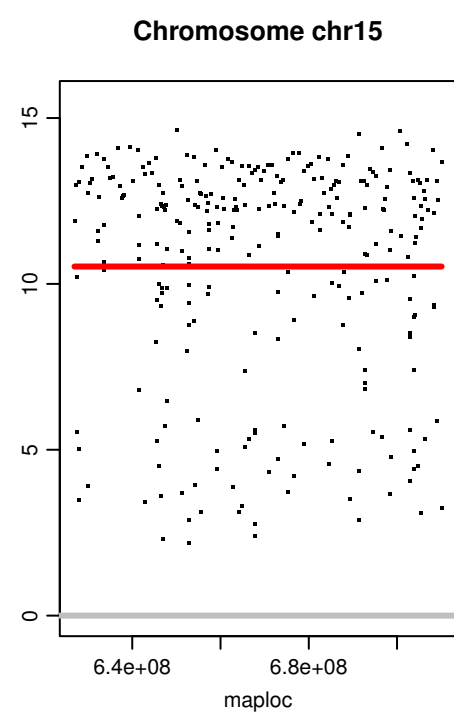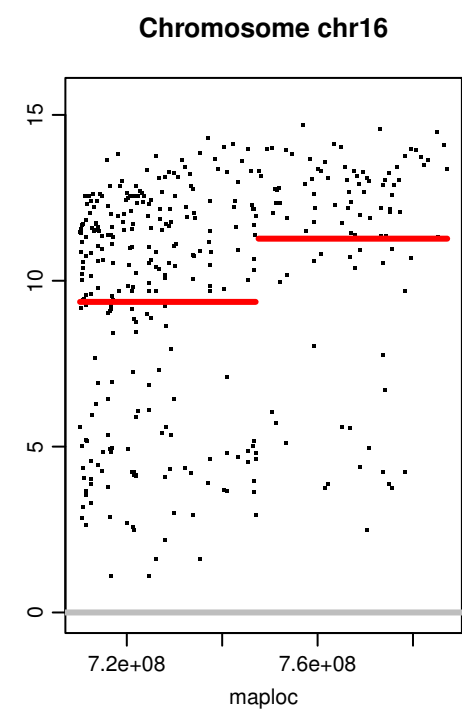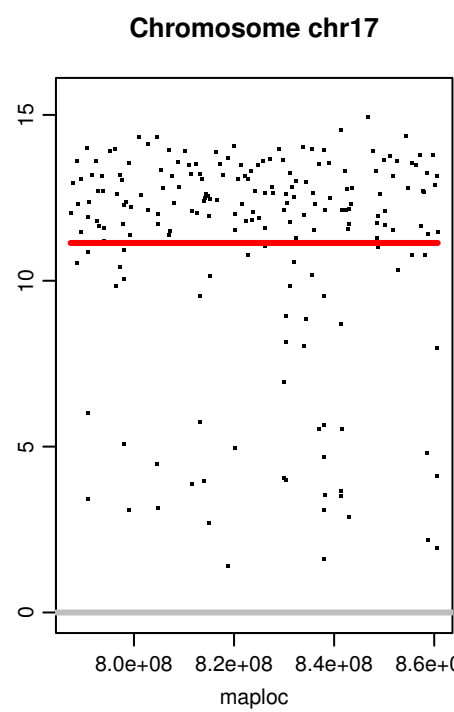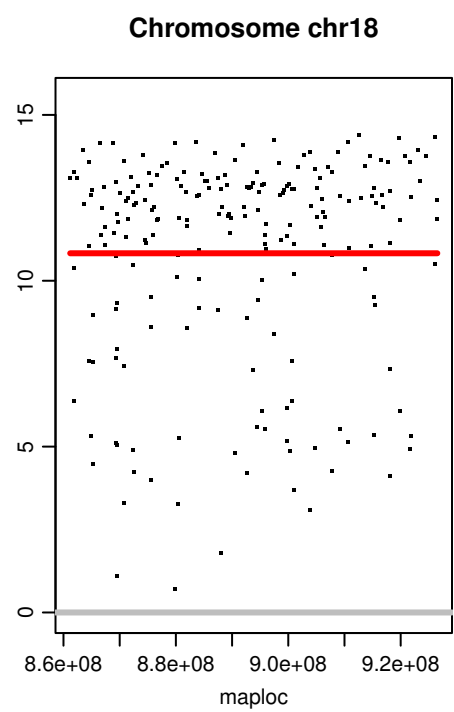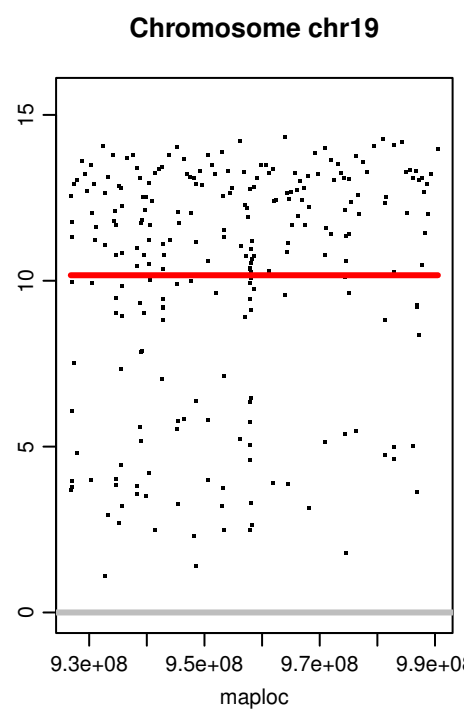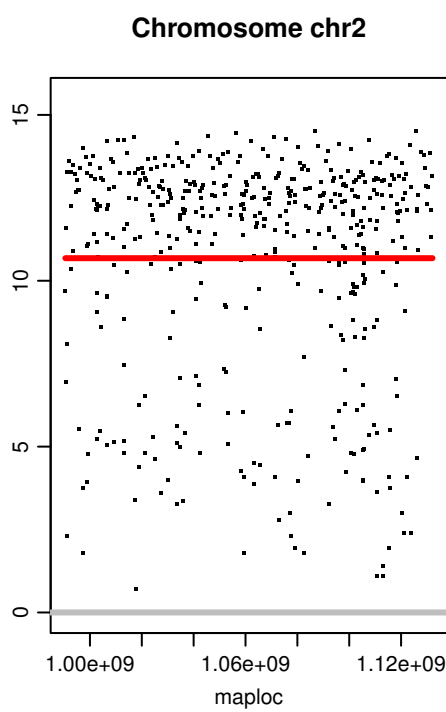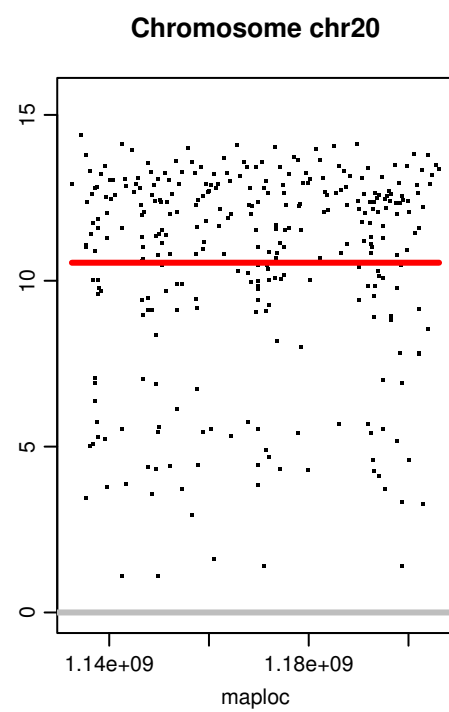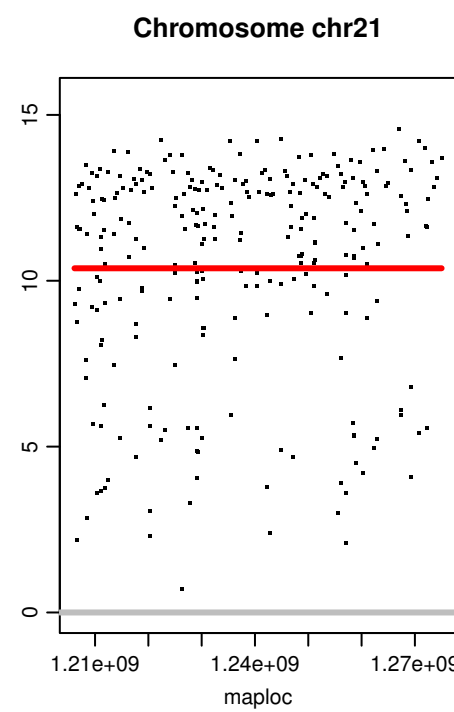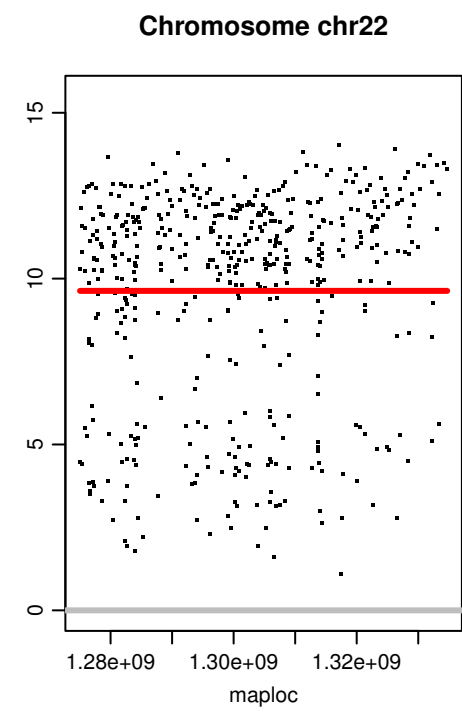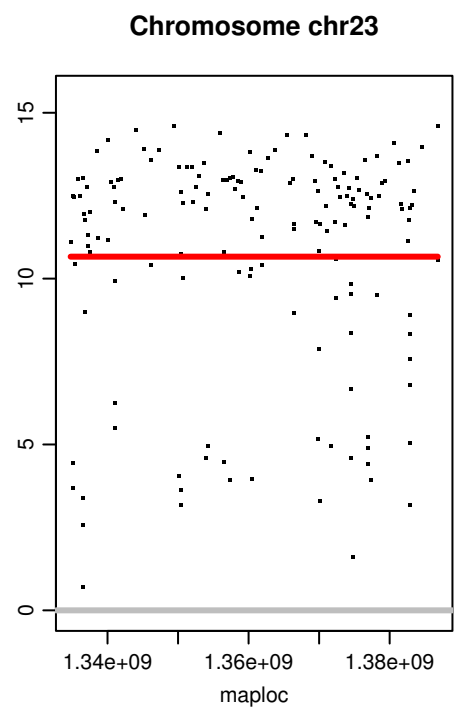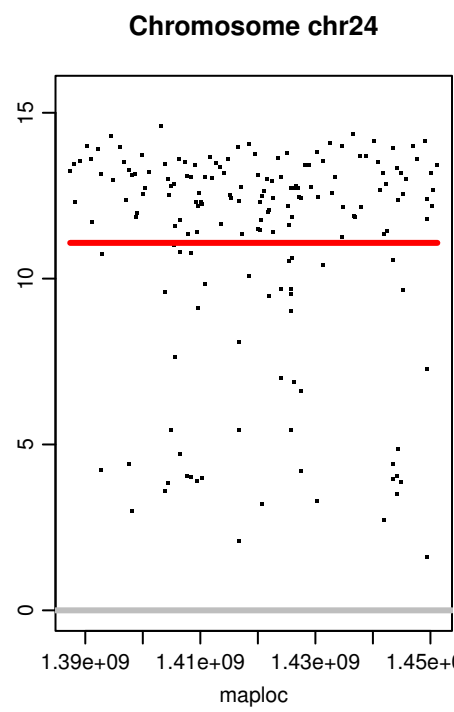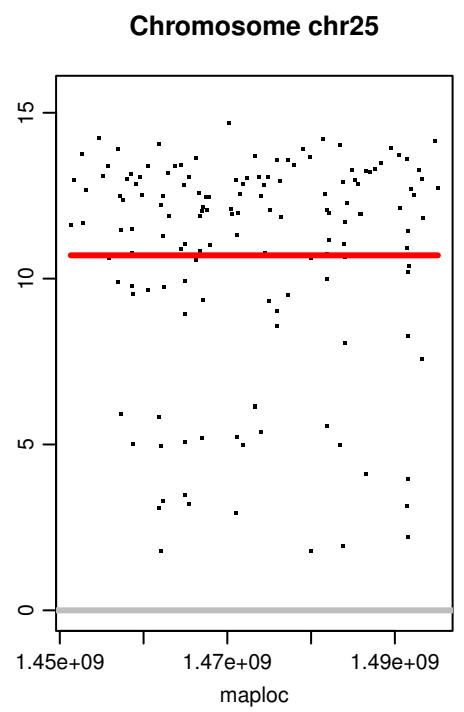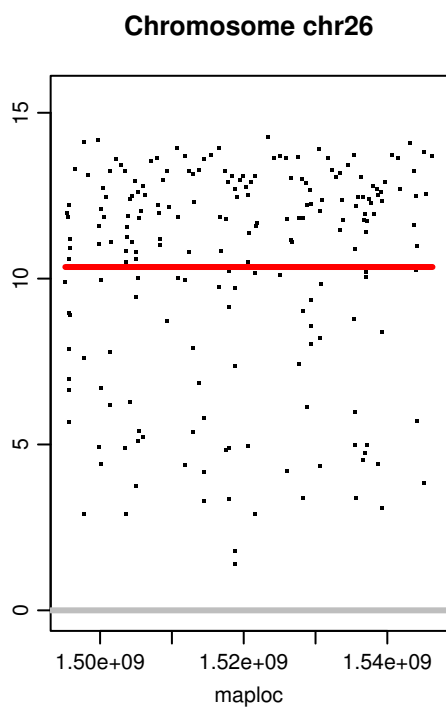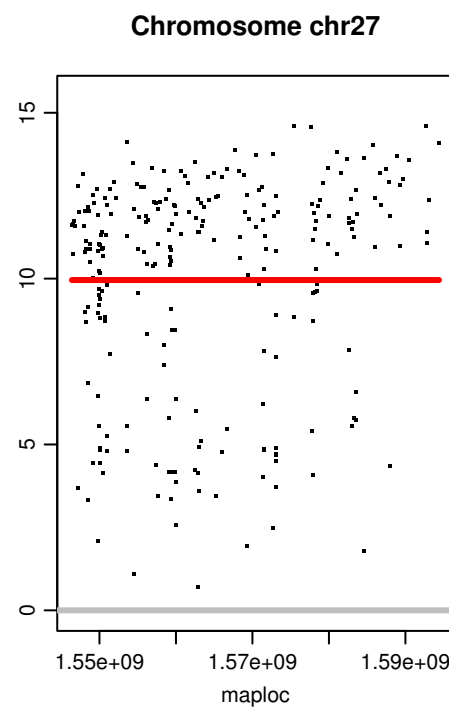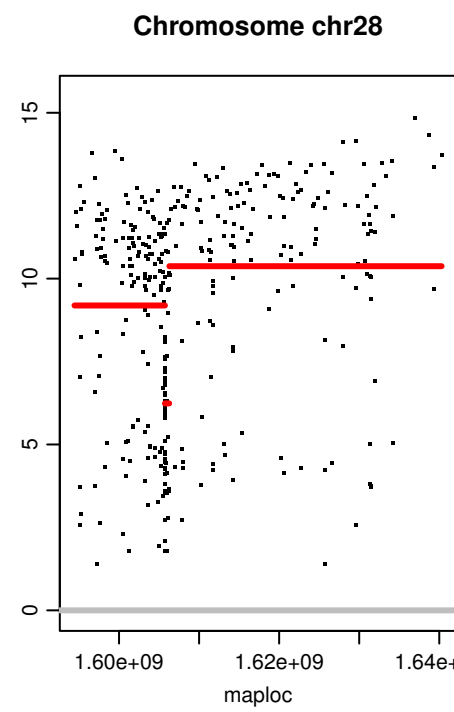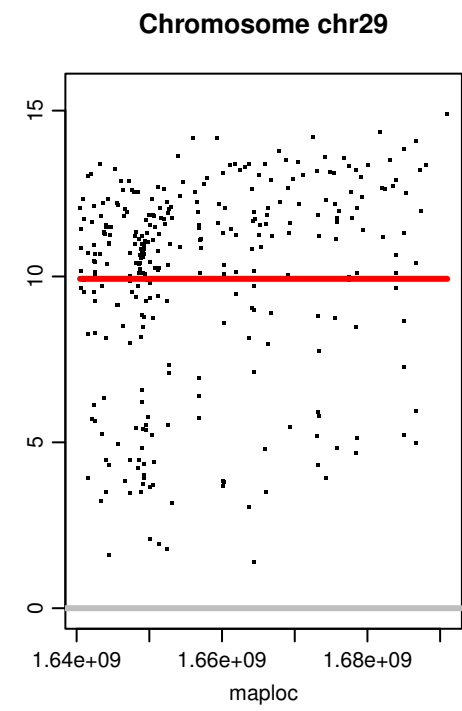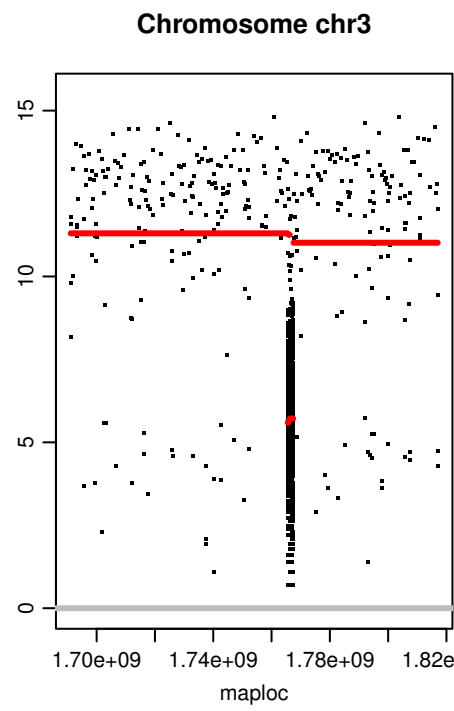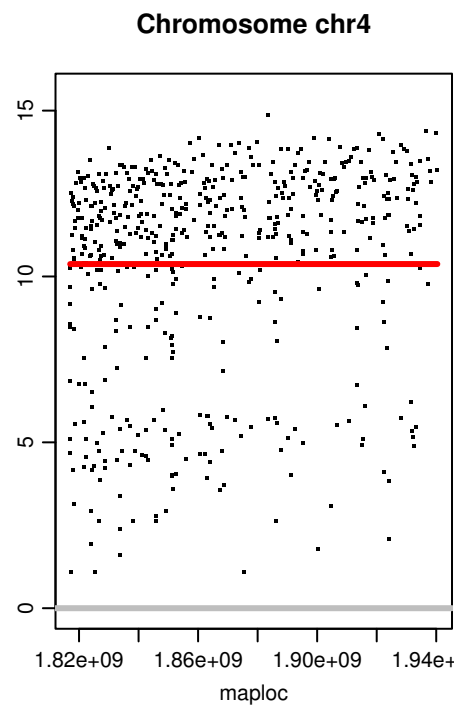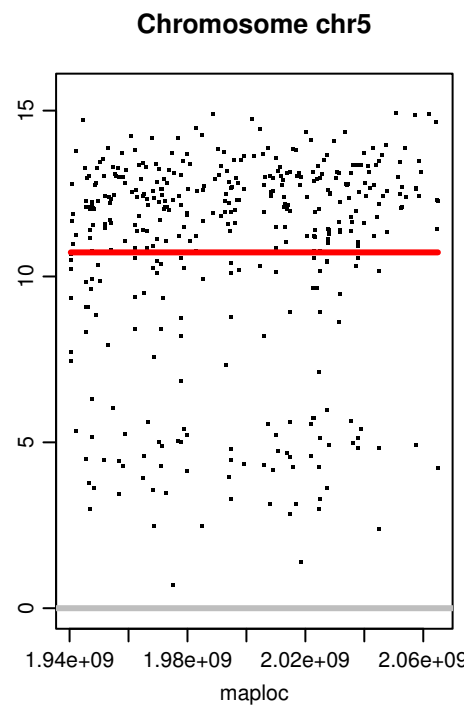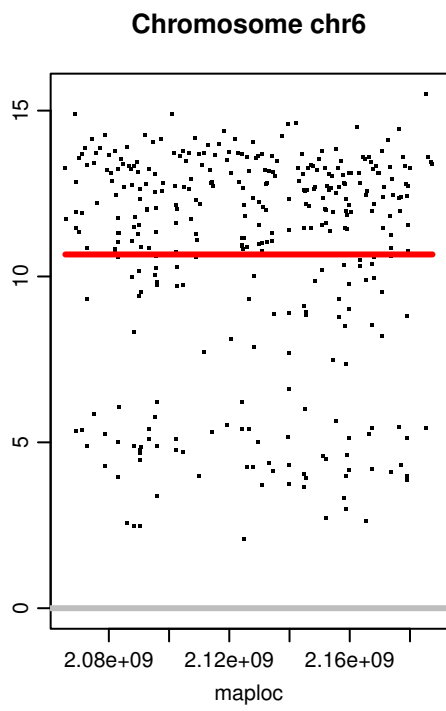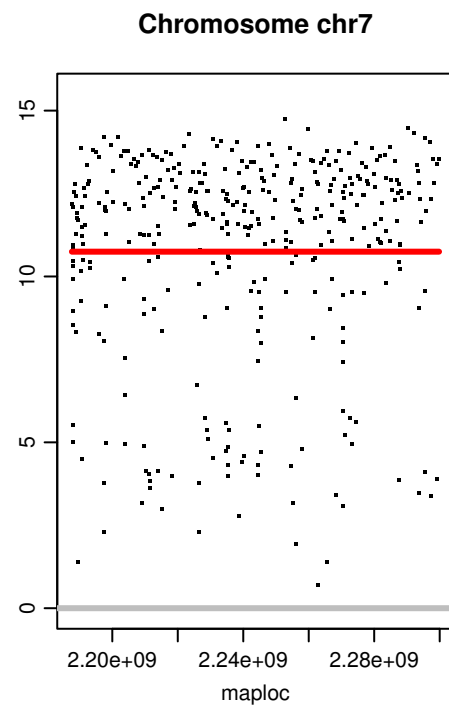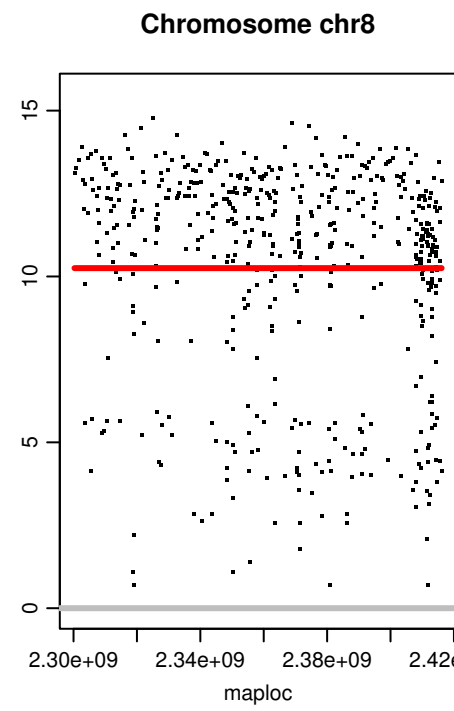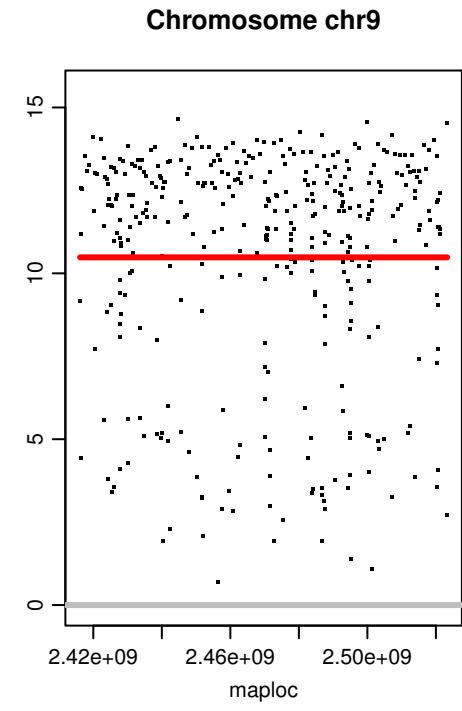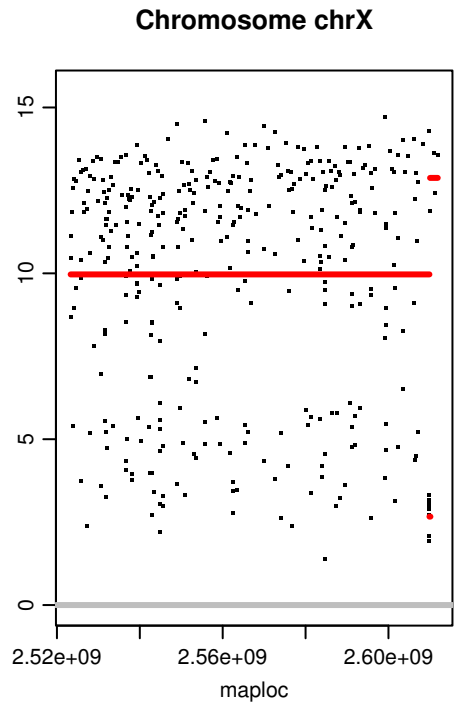

CPYB2

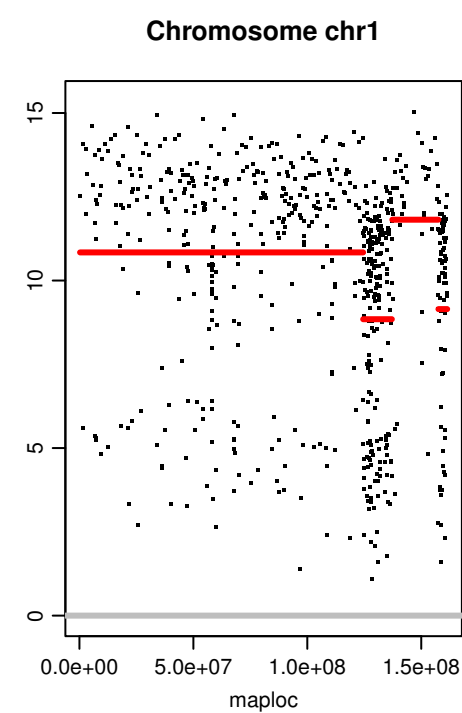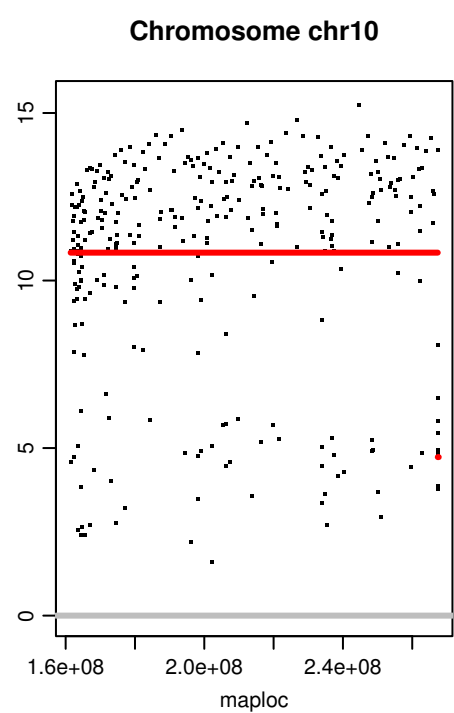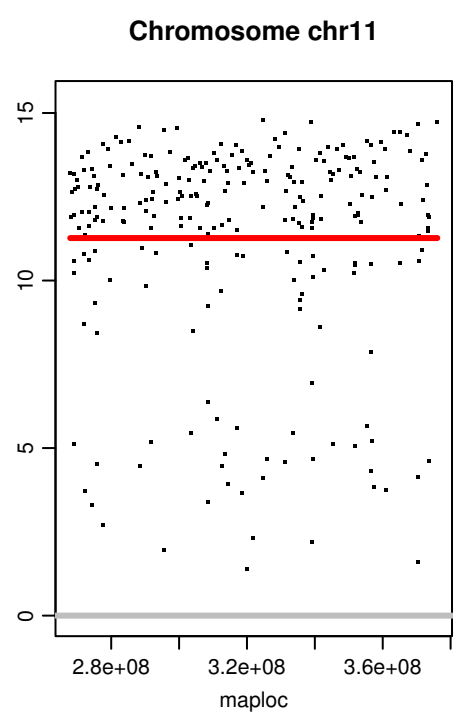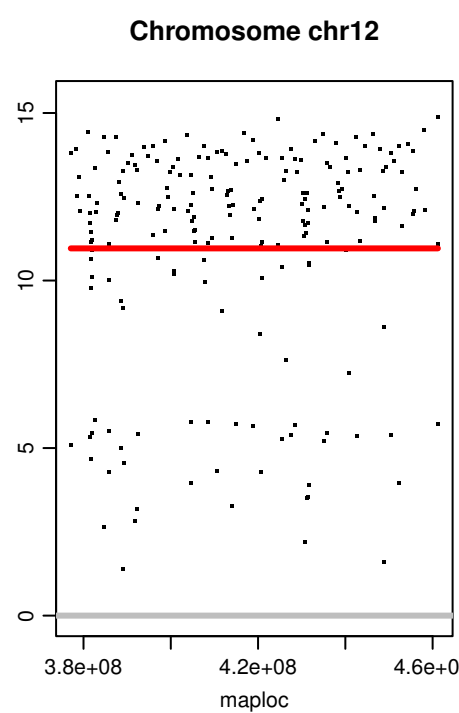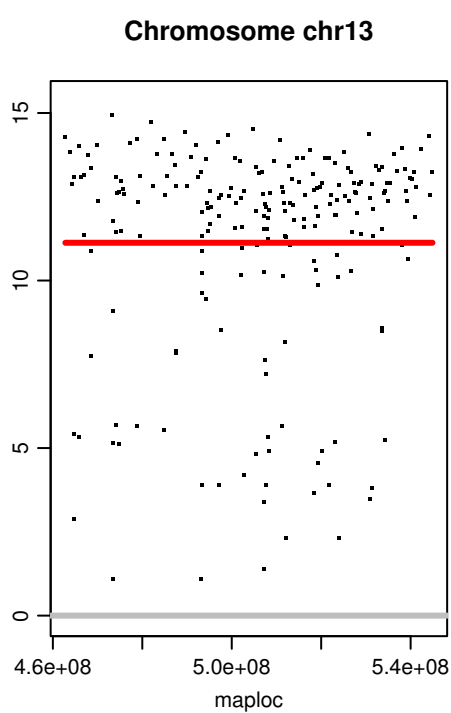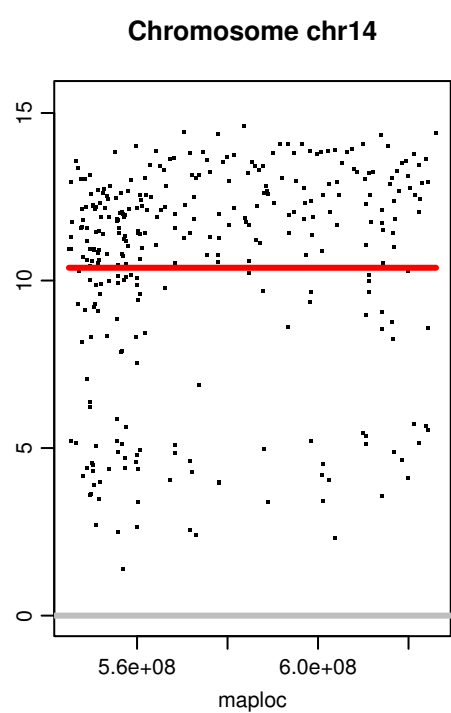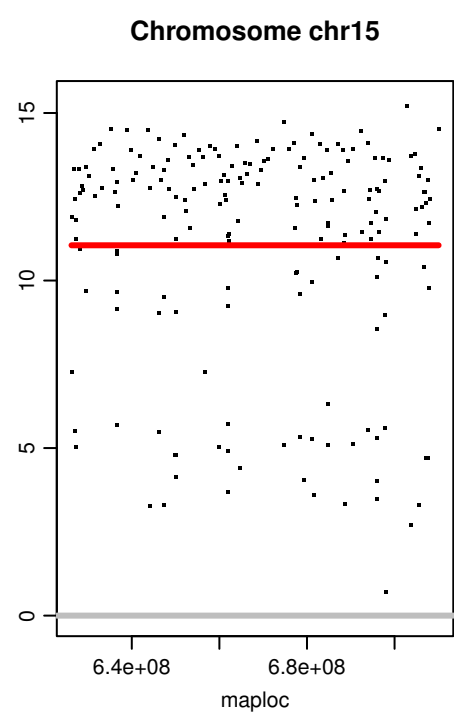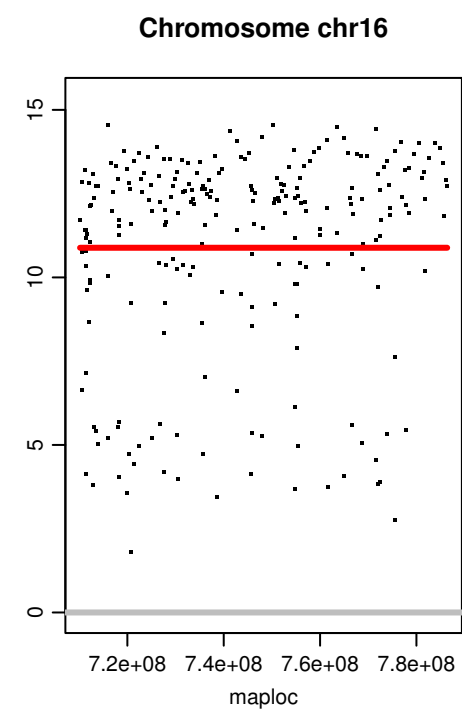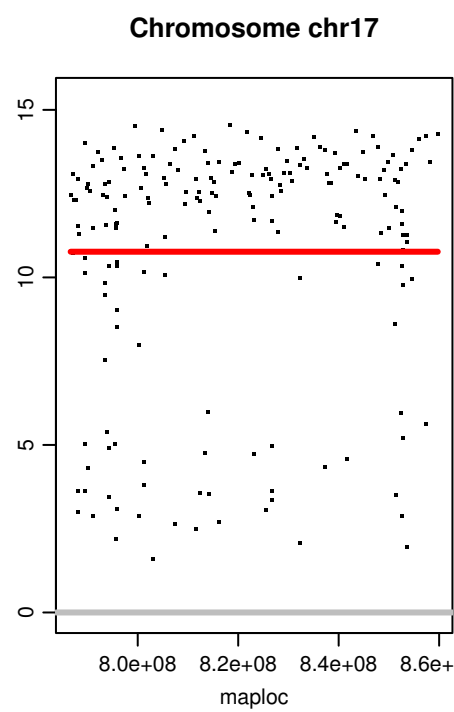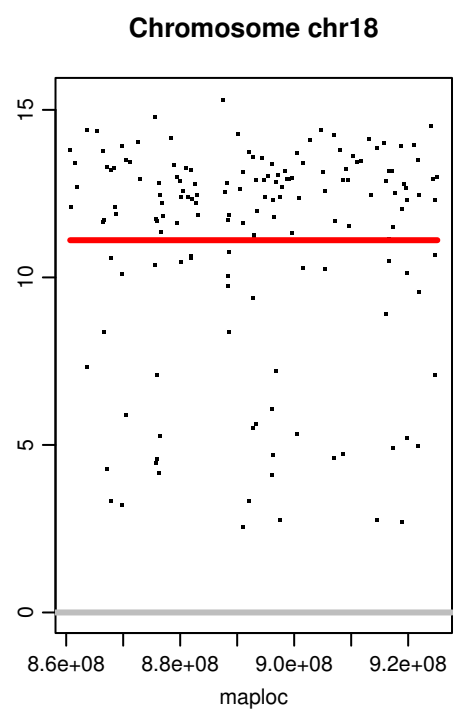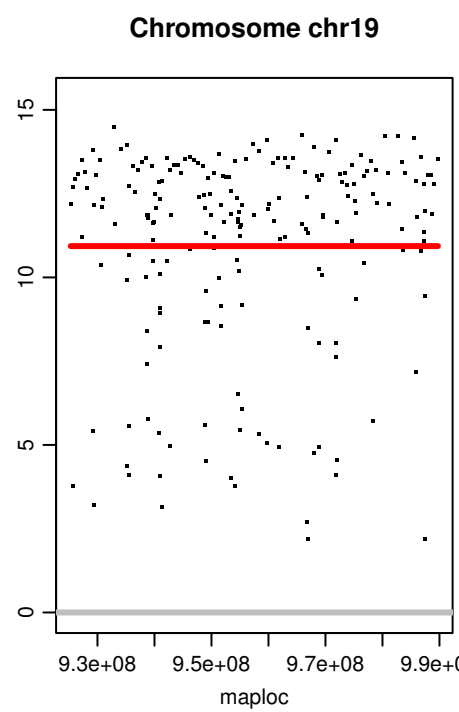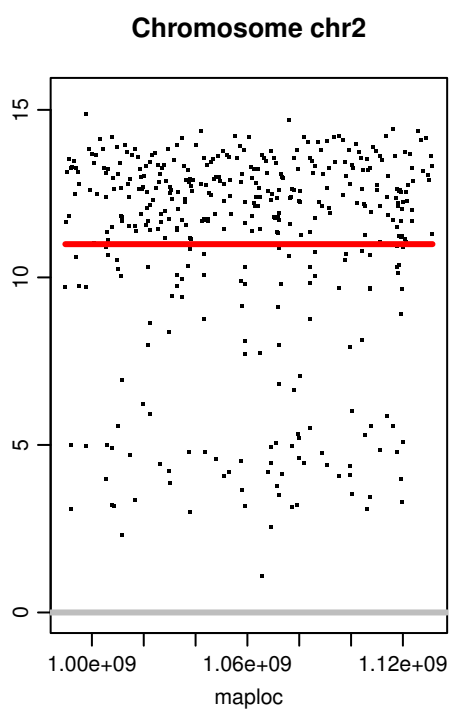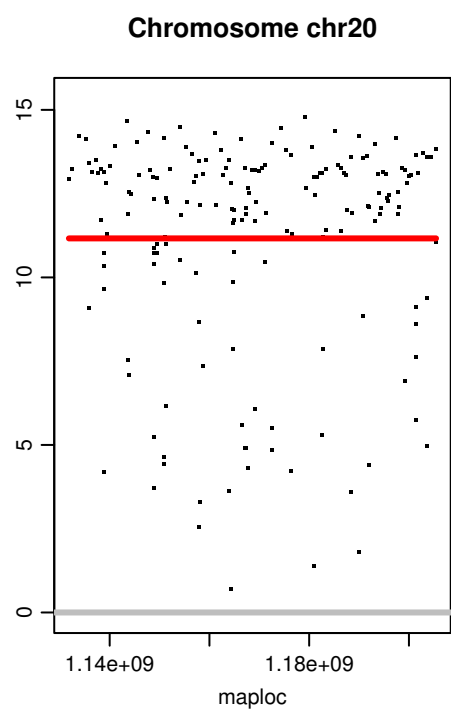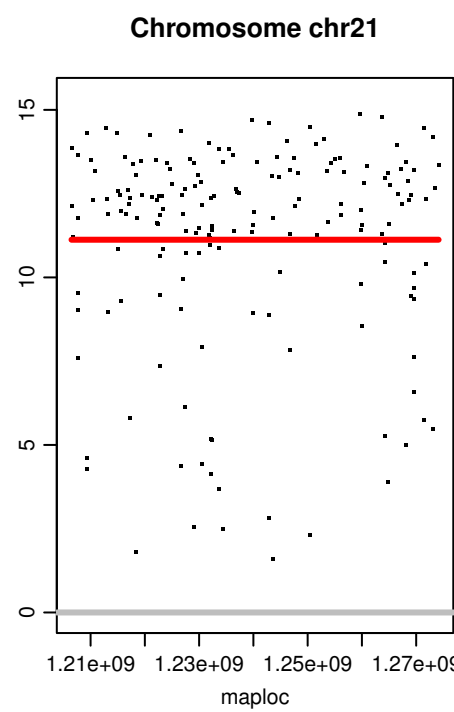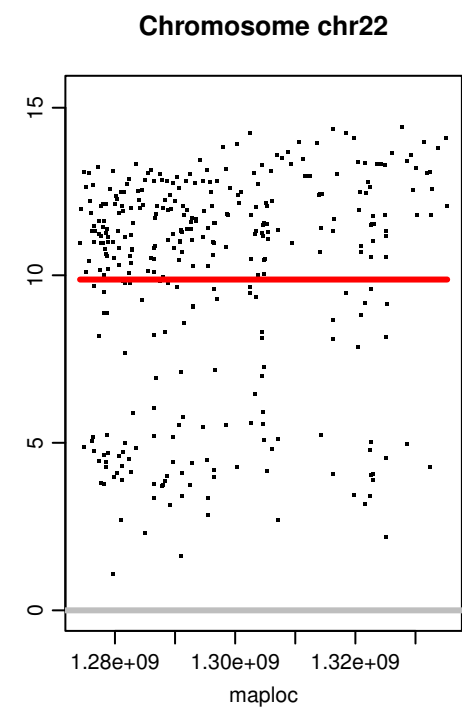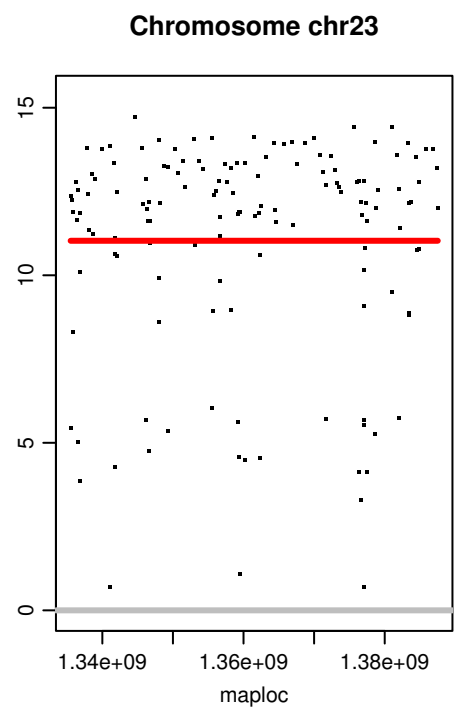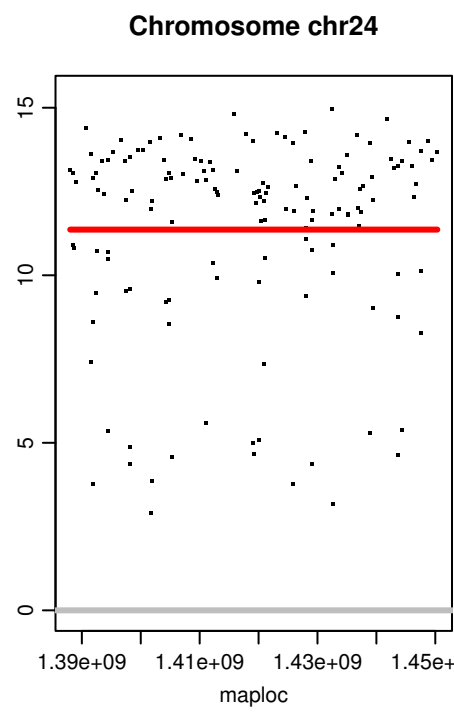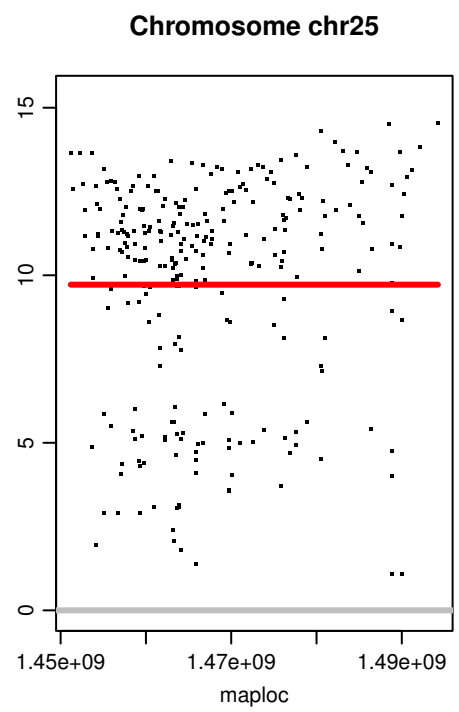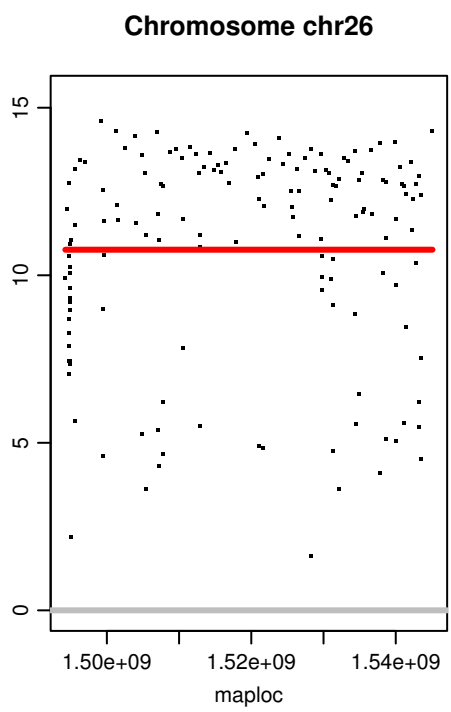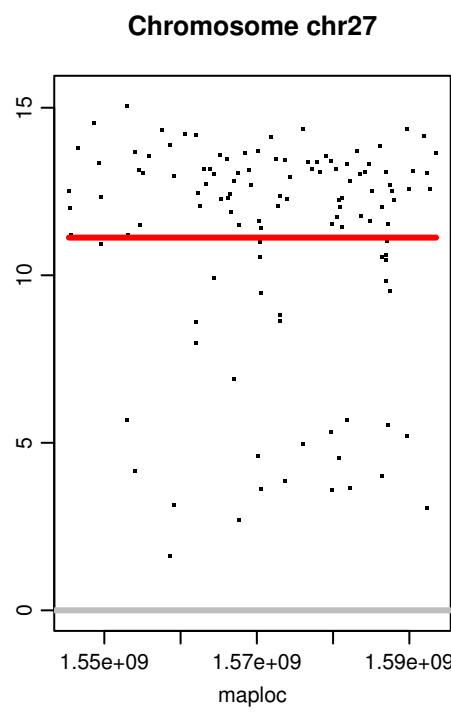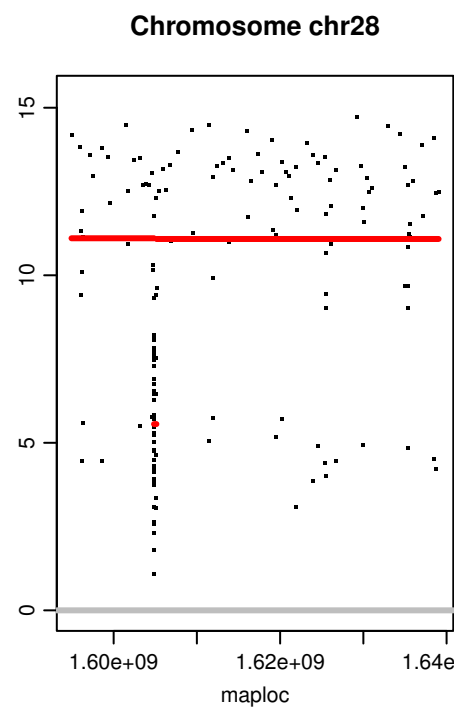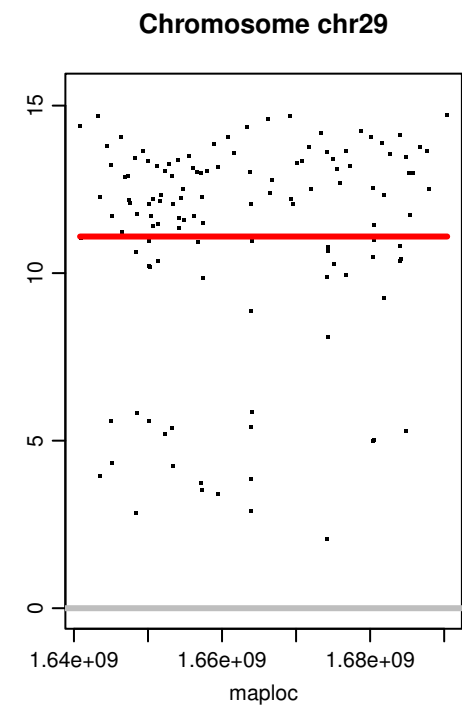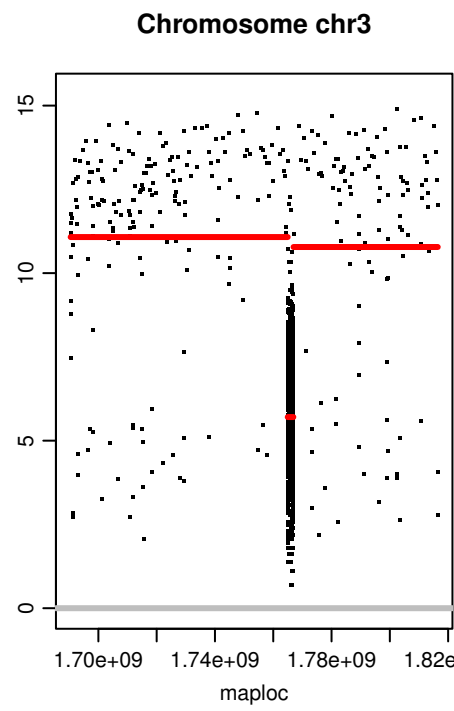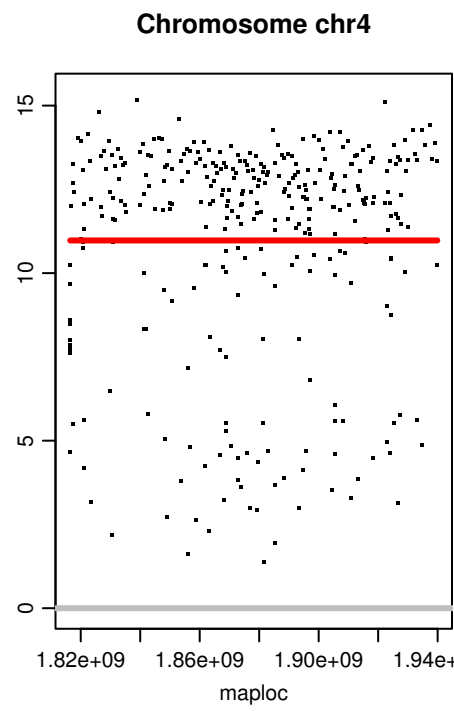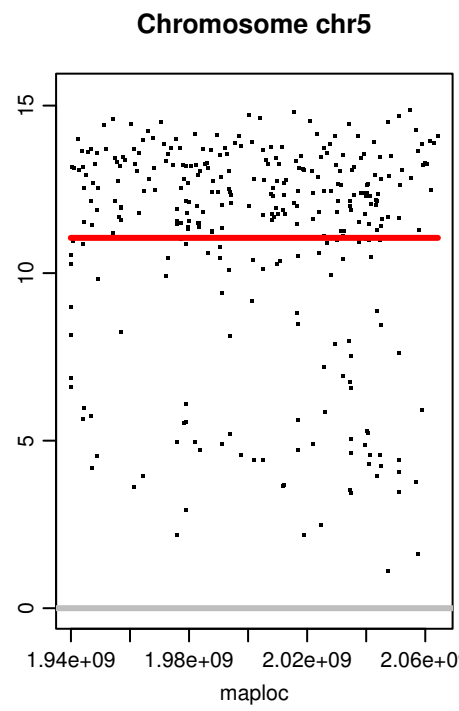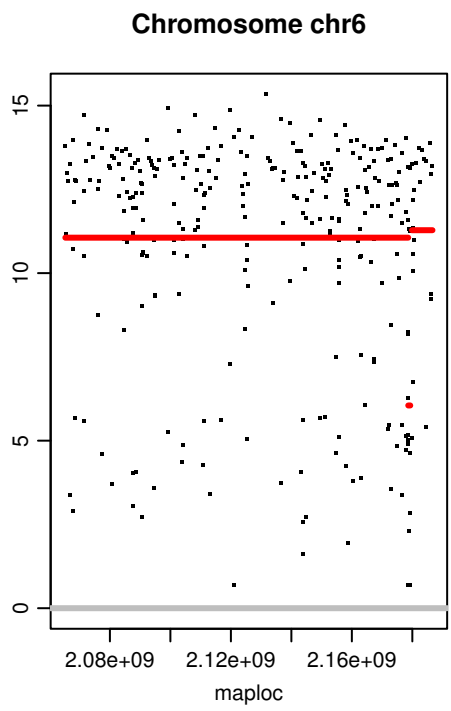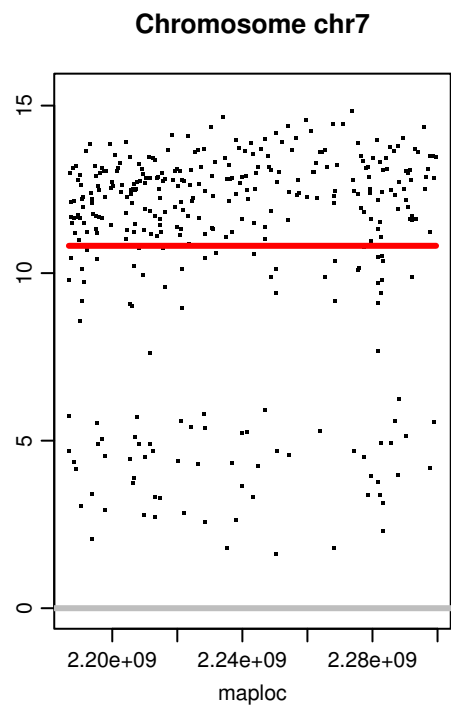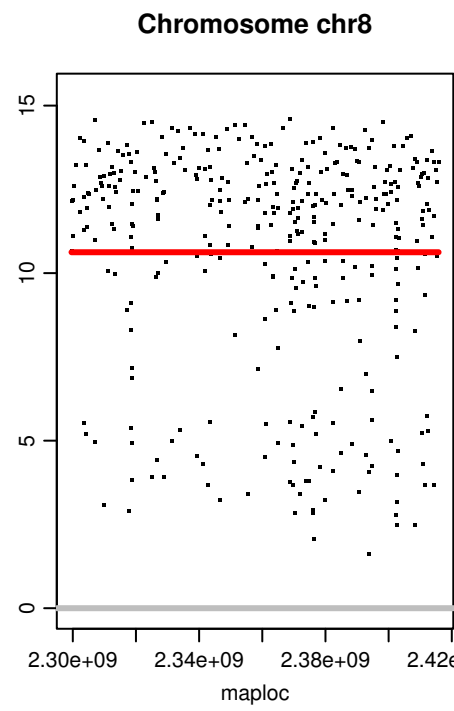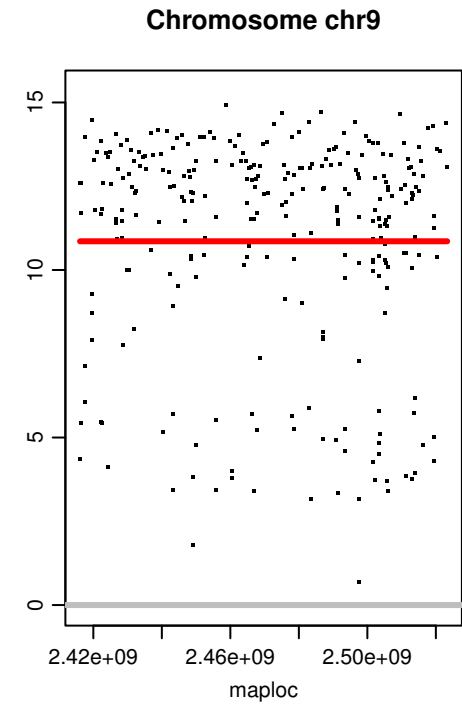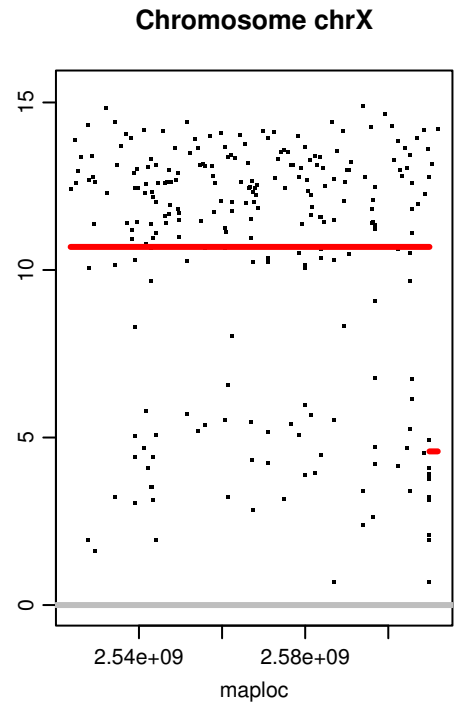

MGOB

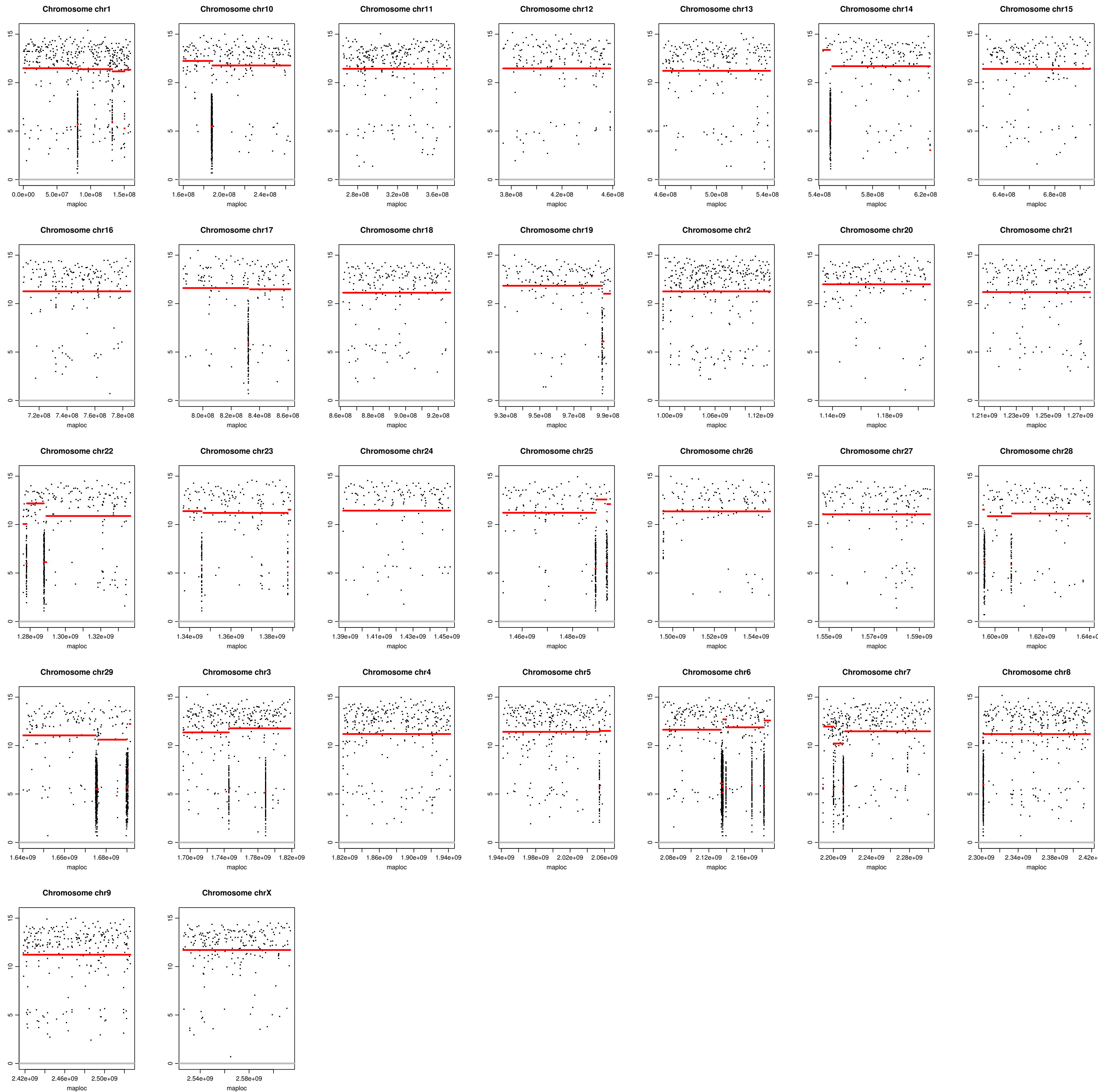

Supplement: Additional file 2: — Distance between positions plots. Plots are presented for all chromosomes of reference genomes (canFam3 for CFA12, bosTau7 for BTAMix, CPYB1, CPYB2, and MGOB). Axes: X – ln(distance between consecutive DOP-positions, bp), Y – cumulative chromosome length, bp. Black dots – individual distances, red lines – mean values. (PDF 958 kb) [file 12864_2016_2933_MOESM2_ESM.pdf]
